# Calling Structural Variants with Confidence from Short-Read Data in Wild Bird Populations

**DOI:** 10.1093/gbe/evae049

**Published:** 2024-03-15

**Authors:** Gabriel David, Alicia Bertolotti, Ryan Layer, Douglas Scofield, Alexander Hayward, Tobias Baril, Hamish A Burnett, Erik Gudmunds, Henrik Jensen, Arild Husby

**Affiliations:** Department of Ecology and Genetics, Evolutionary Biology Centre, Uppsala University, Uppsala, Sweden; School of Biological Sciences, University of Aberdeen, Aberdeen, UK; BioFrontiers Institute, University of Colorado, Boulder, CO, USA; Department of Computer Science, University of Colorado, Boulder, CO, USA; Department of Ecology and Genetics, Evolutionary Biology Centre, Uppsala University, Uppsala, Sweden; Centre for Ecology and Conservation, University of Exeter, Penryn Campus, Penryn, Cornwall, UK; Centre for Ecology and Conservation, University of Exeter, Penryn Campus, Penryn, Cornwall, UK; Centre for Biodiversity Dynamics, Department of Biology, Norwegian University of Science and Technology, Trondheim, Norway; Department of Ecology and Genetics, Evolutionary Biology Centre, Uppsala University, Uppsala, Sweden; Centre for Biodiversity Dynamics, Department of Biology, Norwegian University of Science and Technology, Trondheim, Norway; Department of Ecology and Genetics, Evolutionary Biology Centre, Uppsala University, Uppsala, Sweden

**Keywords:** structural variation, short reads, high-confidence variants, rapid manual curation, curation strategies, putative false positives

## Abstract

Comprehensive characterization of structural variation in natural populations has only become feasible in the last decade. To investigate the population genomic nature of structural variation, reproducible and high-confidence structural variation callsets are first required. We created a population-scale reference of the genome-wide landscape of structural variation across 33 Nordic house sparrows (*Passer domesticus*). To produce a consensus callset across all samples using short-read data, we compare heuristic-based quality filtering and visual curation (Samplot/PlotCritic and Samplot-ML) approaches. We demonstrate that curation of structural variants is important for reducing putative false positives and that the time invested in this step outweighs the potential costs of analyzing short-read–discovered structural variation data sets that include many potential false positives. We find that even a lenient manual curation strategy (e.g. applied by a single curator) can reduce the proportion of putative false positives by up to 80%, thus enriching the proportion of high-confidence variants. Crucially, in applying a lenient manual curation strategy with a single curator, nearly all (>99%) variants rejected as putative false positives were also classified as such by a more stringent curation strategy using three additional curators. Furthermore, variants rejected by manual curation failed to reflect the expected population structure from SNPs, whereas variants passing curation did. Combining heuristic-based quality filtering with rapid manual curation of structural variants in short-read data can therefore become a time- and cost-effective first step for functional and population genomic studies requiring high-confidence structural variation callsets.

**Significance**

Calling and genotyping structural variation with short-read resequencing data has been facilitated by a broad range of bioinformatic tools, but can be fraught with very high false-positive rates. To address this problem, we apply heuristic-based filtering in tandem with rapid manual curation, resulting in significant reduction of putative false positive calls from ∼30% to 80% with the most lenient curation strategy, depending on variant class. Given the substantial reduction in putative false positives for downstream callsets even when applying only minimal manual curation effort, we recommend that detection and genotyping of structural variants for population genomic resequencing studies should be followed by both heuristic-based quality filtering and manual curation, a time- and cost-effective step for enriching callsets with high-confidence variants, i.e. putative true positives.

## Introduction

Structural variants (SVs; e.g. insertions/deletions, inversions, and duplications) have long been recognized as important in evolutionary processes (Sturtevant 1921; Dobzhansky 1937; Noor et al. 2001; Lynch 2007; Fuller et al. 2018). However, characterizing SVs in genomic data has presented an enduring challenge (Cameron et al. 2019; Mahmoud et al. 2019; Bertolotti et al. 2020). Recent technological advancements now make it possible to accurately characterize a broader range of SVs across the genomes of wild organisms. Resulting examples have highlighted the importance of SVs in both evolutionary and conservation-oriented contexts. For example, large-scale inversions play key roles in intraspecific life history polymorphisms in white-throated sparrows (*Zonotrichia albicollis*; Merritt et al. 2020) and ruffs (*Calidris pugnax*; Küpper et al. 2016; Lamichhaney et al. 2016), while small SVs (<100 bp) have also been found to play important roles in adaptation and speciation, for example in cichlid fish (Kratochwil et al. 2019; McGee et al. 2020). On the other hand, deleterious SVs may be overrepresented in small populations (Wold et al. 2021). As such, inquiries into the fitness effects of SVs (Gaut et al. 2018; Zhou et al. 2019) and their relative contribution to mutational load are becoming increasingly important for applied conservation genomics (Wold et al. 2023).

Part of the renewed interest in SVs and their identification is driven by advances in bioinformatic tools that facilitate the detection of SVs in sequencing data. However, considerable challenges still remain related to the incidence of false positives, which are known to far exceed the proportion of true positives—even for SVs discovered using short-read data at recommended (>20×) coverage (Belyeu et al. 2021; Wold et al. 2023). For example, in a recent study on structural variation in 492 Atlantic salmon (resequenced at an average 8.1× coverage with Illumina short reads), Bertolotti et al. (2020) reported that up to 91% of identified SVs were false positives after visual inspection with Samplot (Belyeu et al. 2021). Such high false discovery rates have also been reported elsewhere (Cameron et al. 2019; Kosugi et al. 2019) and highlight dangers of relying on bioinformatic approaches alone to identify SVs, particularly when using short-read sequencing data. While long-read sequencing and chromatin conformation capture for genome assembly and SV detection can help mitigate this problem (Mérot et al. 2020; Sirén et al. 2021; Liao et al. 2023), using short-read mapping approaches will continue to play a prominent role given the low costs involved, the large amounts of short-read data available, and the continued prevalence of study systems possessing only a single reference genome assembly. Furthermore, many studies focusing on wild organisms may be limited in terms of both time and computational allocations, for example to test or compare outputs from multiple short-read SV discovery tools, which tend to be particularly resource hungry (Wold et al. 2023). As such, guidelines for time- and cost-effective identification of SVs from short-read data are of particular value, in order to address the current high false-positive rates and associated risks of misleading downstream analyses.

To address the issue of high false-positive rates, several approaches have been developed, particularly the combined use of multiple tools (“ensemble algorithms”) to try to reduce error rates by intersecting variant calls (Ho et al. 2020) and visual inspection (“manual curation”) of all identified SVs (Bertolotti et al. 2020; Belyeu et al. 2021). Ensemble approaches can still show high false discovery rates (Cameron et al. 2019; Schikora-Tamarit and Gabaldón 2022), while traditional manual curation methods, in, for example, the Integrative Genome Viewer (IGV), can be time consuming, though automation of the curation process has the potential to improve the latter approach substantially (Belyeu et al. 2021). In Bertolotti et al. (2020), the bioinformatic approach using LUMPY/smoove (Layer et al. 2014) identified over 165,000 SVs across all 492 individuals. All SVs were visually inspected by a team of curators, taking 5.73 (8-h) d on average per curator, a reasonable investment given the potential cost incurred by including a substantial proportion of putative false-positive calls.

**Fig. 1.**
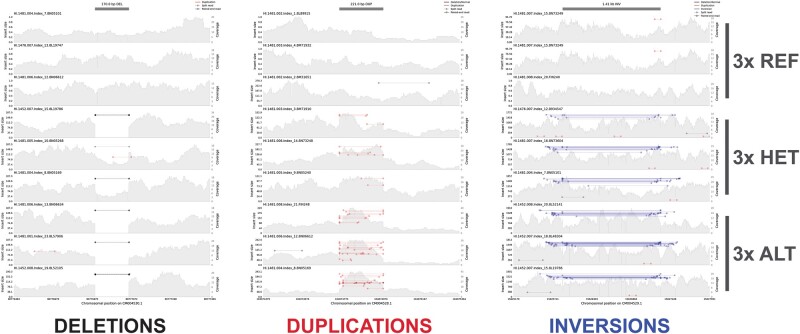
An example layout of Samplot-generated images in PlotCritic. The rapidity and efficiency of manual curation are greatly improved by leveraging the context of individuals with differing genotypes in a fixed order, in this case three homozygote reference individuals, three heterozygote individuals, and three homozygote alternate individuals. Here, three putative true positive SVs are shown, each plotted for nine individuals representing all three genotypes. Note that fewer individuals per genotype may be visualized than shown here, allowing for curation of lower-frequency variants.

Here, to provide insights into the reliability of short-read sequencing data for SV detection, we use whole genome medium-coverage (∼10×) short-read data from Fennoscandian house sparrows (*Passer domesticus*) to examine the structural variation landscape in a species with a relatively compact vertebrate genome size (∼1.3 Gb; Challis et al. 2023). By visualizing different classes of SVs from multiple individuals of the same genotype, we improve upon Bertolotti et al.'s automated method using Samplot/PlotCritic. We increase the efficacy and rapidity of manual visual curation by allowing the curator to contrast expected genotypes in a consistent order (two to three individuals of homozygote wild type, heterozygote, and homozygote alternate for polymorphic variants or only individuals homozygous for wild type or homozygote alternate alleles; Fig. 1). Using this improved manual application of Samplot/PlotCritic, we demonstrate that putative false-positive rates are high in short-read data from a wild bird species and show there is a clear need for visual curation of SVs prior to downstream analyses. We also examine the tradeoff between lenient (e.g. using a single curator) versus stringent curation strategies (e.g. using multiple curators) and investigate to what extent these strategies agree in relative proportions of putative false positives rejected and high-confidence variants retained.

## Results

### Accurate SV Detection

To ensure accurate SV detection, we built upon the strategies recommended by Bertolotti et al. (2020) using a single generalist program followed by automated manual curation. Rather than adopt an “ensemble algorithm” approach by intersecting calls from multiple programs, which has been shown to result in the retention of a substantial proportion of false positives (Cameron et al. 2019; Mahmoud et al. 2019; Wold et al. 2021), we instead used the generalist SV caller LUMPY (Layer et al. 2014) to call larger (>20 bp) SVs (deletions, duplications, and inversions) from aligned short reads. We then genotyped the resulting calls with SVTyper (Chiang et al. 2015) and added annotations for fold-change in sequencing depth for SV calls compared to their flanking regions with Duphold, via the smoove pipeline (Pedersen et al. 2020). This produced an initial population-wide VCF of 15,029 deletions, 3,430 duplications, and 1,188 inversions (Table 1).

**Table 1:** Size range (in bp) for raw, filtered, and curated SV classes

Curation	Maximum length	Minimum length	Mean length	Median length	Count
Deletions					
Raw, uncurated	142,501,103	23	319,153	376	15,029
Samplot-ML	142,501,103	23	185,764	347	14,345
Duphold	81,855,813	23	110,402	333	13,894
One curator (lenient)	6,096	25	257	106	2,457
All four curators	6,096	25	260	109	1,243
Duplications					
Raw	120,312,679	79	1,497,613	1350	3,430
Duphold	120,312,679	79	917,802	625	2,560
One curator (lenient)	2,121,873	95	13,290	345	287
All four curators	411	98	162	126	37
Inversions					
Raw, uncurated	76,386,443	33	639,090	83	1,188
One curator (lenient)	2,057	37	87	376	177
All four curators	107	49	68	63	13

Counts for one curator (the most lenient curator; strategy rejecting the fewest variants) and four curator callsets are fractions of the subset filtered by “genotype frequency” (SVs selected for curation that are represented by at least three individuals of each genotype class; see supplementary table S1, Supplementary Material online). Samplot-ML only applicable for deletions; Duphold (filtering by fold-change in variant coverage relative to flanking regions) only for deletions and duplications.

**Fig. 2.**
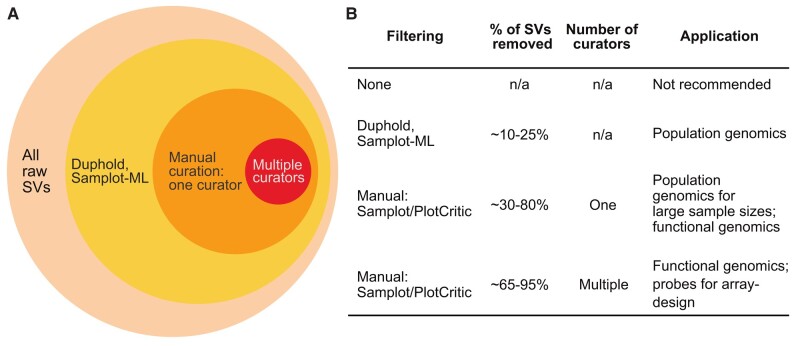
a) The tradeoff between the proportion of rejected SVs (deletions, duplications, and inversions) following heuristic-based filtering and manual curation versus the increasing confidence that SVs may represent putative true positives. The proportion of retained SVs after each filtering step is represented by circles; smaller circles indicate a decreasing number of retained SVs, coupled with increasing confidence in SV calls and genotypes. b) Summary of filtering methods, proportion of SVs removed, number of manual curators required, and downstream applications for SV callsets of varying confidence. Percentages for one and four curator callsets are fractions of the “genotype frequency filtered” subset (SVs selected for curation that are represented by at least three individuals of each genotype class). Note that automated curation with Samplot-ML is currently only possible for deletions.

As recently recommended by Wold et al. (2023), we then filtered raw deletions and duplications based on call quality, using the Duphold annotation “DHFFC” (“duphold flank fold-change”). DHFFC is a heuristic metric quantifying the degree of fold-change reasonably expected in regions flanking a putative true positive deletion or duplication; it is therefore not applicable for inversions (Pedersen and Quinlan 2019). Duphold (call quality) filtering rejected 7.6% of raw deletions (filtered for DHFFC < 0.7) and 25.4% of raw duplications (filtered for DHFFC > 1.3; see Materials and Methods) as putative true positives for downstream manual curation (Fig. 2b and Table 2). As recommended by Wold et al. (2023), call quality filtering with Duphold was followed by genotype quality filtering of individual genotypes, based on Mean Smoove Heterozygote Quality annotations (MSHQ). MSHQ (genotype quality) filtering rejected 9% of deletions, 41% of duplications, and 8% of inversions.

**Table 2:** Relative proportions (%) of putative false-positive variants rejected by each filtering step

Filtering method	>20 to 100 bp	>100 to 250 bp	>250 to 500 bp	>500 bp to 1 kb	>1 to 5 kb	>5 to 10 kb	>10 to 500 kb	>500 kb	Percent rejected (total variants removed)
Deletions									
Samplot-ML	0.4	0.6	1.9	2.5	6.6	6.3	27.5	45.5	4.6 (684)
Duphold	0.8	2.5	4.0	5.5	11.4	6.7	35.9	67.0	7.6 (1,135)
One curator (lenient)	7.9	12.2	47.8	58.6	56.1	96.0	100.0	100.0	29.0 (1,004)
All four curators	54.4	51.1	77.1	80.1	76.0	98.0	100.0	100.0	64.1 (2,218)
Duplications									
Duphold	0.0	0.9	5.4	20.6	32.5	48.8	47.0	41.9	25.4 (870)
One curator (lenient)	50.0	47.7	70.8	87.3	59.5	93.9	99.0	98.9	77.6 (2,149)
All four curators	90.0	85.6	97.8	100.0	100.0	100.0	100.0	100.0	97.1 (3,393)
Inversions									
One curator (lenient)	23.9	30.8	50.0	66.7	0.0	100.0	100.0	100.0	30.0 (75)
All four curators	94.4	92.3	100.0	100.0	100.0	100.0	100.0	100.0	94.8 (239)

Percentages for one curator (the most lenient curator; strategy rejecting the fewest variants) and four curator callsets are fractions of the subset filtered by “genotype frequency” (SVs selected for curation that are represented by at least three individuals of each genotype class; see supplementary table S1, Supplementary Material online). Samplot-ML only applicable for deletions; Duphold (filtering by fold-change in variant coverage) only for deletions and duplications.

### Evaluating Alternative Strategies for Rapid Manual SV Curation

To build upon the recommendations of Bertolotti et al. (2020) for manual curation speed and efficiency, we evaluated curation performance of SVs using both deep learning and single versus multiple human curators (Fig. 2a). We first applied Samplot-ML on the full deletion callset, a pipeline for automated curation by deep learning, currently only available for deletions (Belyeu et al. 2021). This step rejected ∼5% of all raw deletions, supporting earlier insights from Belyeu et al. (2021) that Samplot-ML removes similar proportions of deletions as Duphold (call quality) filtering (Fig. 2b and Tables 1 and 2).

To evaluate the speed and efficiency of removing putative false positives through automated manual curation, we compared curation performance between single and multiple curators by applying the curation approach demonstrated and validated in Bertolotti et al. (2020) and Belyeu et al. (2021). To ease manual curation, we chose to only examine SVs represented by a minimum of three individuals per genotype class (i.e. three homozygote references, three homozygote alternates, and three heterozygotes, hereafter referred to as the “genotype frequency filtered” subset) using Samplot and PlotCritic (formerly referred to as SV-Plaudit; Belyeu et al. 2018, 2021). Filtering by this genotype frequency threshold removed 77% of all raw deletions (11,568 removed), 62.7% of all raw duplications (2,149 removed), and 78.8% of all raw inversions (936 removed; supplementary table S1, Supplementary Material online), retaining a total of 3,461 deletions, 1,281 duplications, and 252 inversions for manual curation. We then randomly sampled (with replacement) and plotted two to three individuals of the resulting genotype class for each resulting SV in Samplot (see Fig. 1 for an example of the Samplot layout used in this study; supplementary fig. S2, Supplementary Material online, for a putative true positive deletion; supplementary fig. S3, Supplementary Material online, for a putative false positive inversion; further examples in guidelines for identifying SVs in Supplementary material), using the PlotCritic interface to record curators’ alternative answers to the question: “Is this a real SV?”: “Yes,” “Maybe,” or “No” (see supplementary fig. S1, Supplementary Material online, for an example screenshot of the PlotCritic interface). The “Maybe” category allowed for more rapid curation by reducing time evaluating more ambiguous borderline cases while allowing curators to focus on primarily removing obvious putative false positives. In this case, we chose to consider calls scored as either “Yes” or “Maybe” as putative true positive calls in downstream analyses, but a more stringent callset could be easily created by extracting only “Yes” scores from PlotCritic reports. Each separate curator independently examined the “genotype frequency filtered” subset, comprising a total of 4,994 SV images (3,461 deletions, 1,281 duplications, and 252 inversions), spending an average of 3 to 5 s per image, amounting to only ∼4.2 to 6.9 h of total curation time per person.

**Fig. 3.**
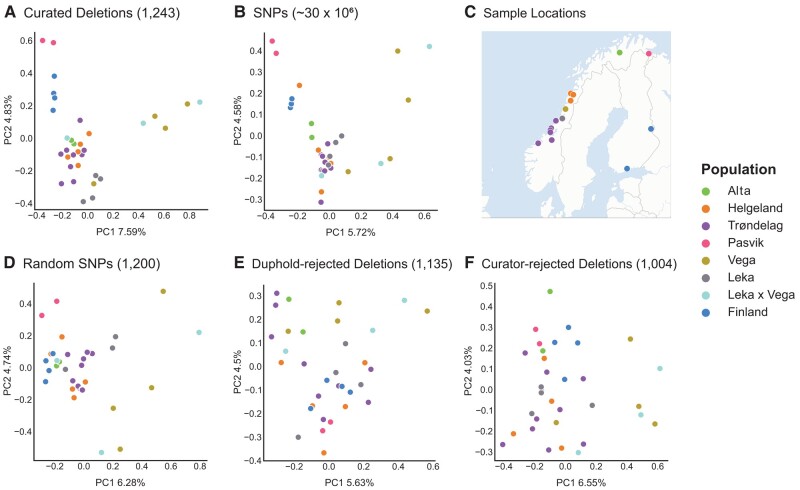
PCAs showing the effect of manual curation with Samplot/PlotCritic and filtering with Duphold on raw SV calls in recapturing expected population structure. a) A total of 1,243 deletions retained by complete agreement between all four curators (strategy minimizing the false-positive rate; least number of putative false positives retained); b) all 30,275,406 raw SNPs; c) sampling locations for all 33 individuals; d) 1,200 randomly downsampled SNPs; e) all 1,135 deletions rejected by Duphold filtering (DHFFC <0.7); and f) all 1,004 deletions rejected by the most lenient curator (strategy minimizing the false-negative rate; least number of putative true positives rejected). Rejected deletions shown in f) were also rejected by near-complete (>99%) agreement between all four curators.

Variation in the total number of rejected putative false-positive SVs was observed between curators and is helpful to inform future standardization of curation strategies. Firstly, this variation probably reflects differences in curation approach between curators, despite similar search images for putative true positive (high-confidence) SVs (see guidelines used to train curators for identifying SVs in Supplementary material). This may occur where a lenient curation strategy is defined as focusing on the removal of obvious putative false positives from a callset rather than attempting to unambiguously identify true positive calls while requiring that all individuals in a given Samplot showed correct genotypes. Only three curators (G.D., H.A.B., and E.G.) used the “Maybe” category, while the most stringent curator (A.B.) did not (only answering “Yes” or “No”), allowing for comparison of individual variation in curation stringency. This further restricted the final callset of the most stringent curator, because “Yes” and “Maybe” calls were all merged and considered downstream as putative true positive (high-confidence) SVs. For callsets curated by the most lenient curator (G.D.), the putative false-positive rate was highest for duplications (78% rejected), but substantially lower for deletions (29% rejected) and inversions (30% rejected; Table 2; supplementary table S2, Supplementary Material online). In contrast, of the variants retained as putative true positive (high-confidence) variants after the intersection of all four curator callsets, the putative false-positive rate was much higher for both duplications (97% rejected) and inversions (95% rejected), compared to deletions (64% rejected; Table 2; supplementary table S2, Supplementary Material online). Variants retained by the most stringent curator (A.B.) were largely a subset of the high-confidence variants retained by all the other curators. Most importantly, >99% of variants rejected as putative false positives by a single, lenient curator (2,073) were also rejected by all other three independent curators (2,065). This demonstrates near-complete agreement between the most lenient and stringent curation strategies in terms of rejection of obvious putative false-positive SV calls. In addition, putative false-positive deletions rejected by both a single curator applying a lenient strategy and all four curators (stringent strategy) do not appear to show significant population structure (Fig. 3f; supplementary fig. S13 and table S3E, Supplementary Material online) compared to curated deletions (Fig. 3a; supplementary table S3A and fig. S7, Supplementary Material online).

High-confidence variants passing manual curation were largely subsets of variants retained by call quality (Duphold) and genotype quality (MSHQ) filtering alone: all but two variants retained by the intersection of all four curator callsets (the most stringent callset) passed both call/genotype quality filtering: 1,242/1,243 deletions, 36/37 duplications, and all inversions (13/13). In this sense, genotype frequency filtering, stringent curation, and/or the addition of successive curators essentially performs as both heuristic call/genotype quality filtering alone, though possibly with substantially fewer putative true positive (high-confidence) SVs retained than with a single, lenient curator. For example, all but one deletion retained by the most lenient curator (G.D.) as putative true positives (2,456/2,457) were also retained by both Duphold filtering and Samplot-ML, while 91% of duplications (262/287) were kept by Duphold filtering. Additional genotype quality filtering with MSHQ on variants retained by the most lenient curator (G.D.) kept 99.9% (2,454/2,457) of deletions, 81% (231/287) of duplications, and 98% (174/177) of inversions. That ∼20% of all duplications retained by the single, lenient curator were rejected by call/genotype quality filtering may be attributed to the fact that G.D.'s curation strategy prioritized the removal of obvious putative false-positive SVs (in contrast to the most stringent curation strategy) while also allowing for occasional individual genotypes to be incorrect for a given Samplot image (in contrast to genotype quality filtering). Given that the average coverage for short-read data used in this study was ∼10×, it may be reasonable to assume that duplications would be especially prone to higher genotyping errors. This could lead to the rejection of potential putative true positive (high-confidence) variants by genotype quality filtering (e.g. with MSHQ) alone, in low-to-medium (<20×) coverage data.

Creation of a “genotyped frequency filtered” subset of variants selected for curation (whereby only common SVs represented by at least three individuals per genotype class were considered for curation) essentially functioned as a form of indirect filtering for minor allele frequency and size. In removing rarer variants by selecting only common SVs for curation, the proportion of larger (>500 bp) SVs was also substantially reduced, without the application of hard size cutoffs (supplementary table S1, Supplementary Material online). As expected, larger variants called by smoove thus do appear to rarer than our genotype frequency threshold. However, not all larger variants were removed by genotype frequency filtering: while most (>90%) but not all deletions and inversions larger than 500 kb were removed, only ∼65% of duplications larger than 500 kb were removed (supplementary table S1, Supplementary Material online). Therefore, stringent filtering by genotype frequency did not substantially change the maximum size classes of variants to be curated (supplementary table S1, Supplementary Material online), relative to raw callsets.

**Fig. 4.**
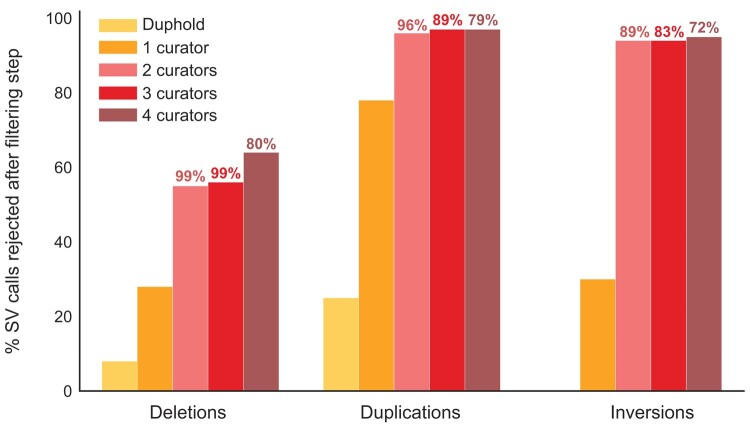
Percent SV calls rejected after each filtering step per SV class. Values above the bar plots indicate the percent “agreement” between all other curators versus the most stringent curator: the intersect between variants rejected by the most stringent curator versus those rejected by other curators. Curators are added in increasing order of stringency, where 1 curator = the most lenient curator (rejecting the fewest variants) and 4 curators includes the most stringent curator (rejecting the most variants). Percent rejected SV calls for one and four curator callsets are fractions of the “genotype frequency filtered” subset (SVs selected for curation that are represented by at least three individuals of each genotype class). Note: Duphold (filtering by fold-change in variant coverage) only applicable for deletions and duplications.

Manual curation further reduced the number and relative proportions of retained SVs of different size classes, relative to raw (uncurated), Samplot-ML–filtered and Duphold-filtered (call quality filtered) callsets (Tables 1 and 2; supplementary table S1, Supplementary Material online), though less markedly when applying only the most lenient curator (G.D.; Tables 1 and 2 and Figs. 2 and 4). More than twice the total number of putative true positive (high-confidence) SVs were retained by a single, lenient curator (2,921 SVs retained) relative to all four curators (1,293 SVs retained; Table 1; supplementary table S2, Supplementary Material online). Putative true positive inversions and duplications retained by a single curator were nearly ten times more numerous than those retained by all four curators, when contrasting the most lenient strategy (single, lenient curator, using both “Yes” and “Maybe” scores) versus the most stringent strategy (only calls retained after intersection between all four curators, including the strictest curator whom only used “Yes” scores). Following curation by all four curators, we observed a marked reduction in the reported maximum length of variants for each SV class as well as a reduction in the median length for deletions and duplications. Both the maximum and median lengths of retained inversions and duplications were markedly higher for a single curator than for all four curators, but not for deletions. The size distributions of retained deletions were similar between single and multiple curators (Table 2; supplementary fig. S9, Supplementary Material online), though almost double the number of larger deletions (from 1 to 5 kb) were retained by a single curator (supplementary fig. S10, Supplementary Material online). However, no duplications or inversions >1 kb were retained by all four curators, while a single curator retained 76 duplications (from 1,011 bp to 2.1 Mb) and inversions (1,406 and 2,057 bp) > 500 bp in length (Table 2; supplementary figs. S11 and S12, Supplementary Material online). Only four putatively true positive SVs exceeded 10 kb in size, all of which were duplications identified by a single curator, ranging from ∼1 kb to 2.1 Mb. In contrast, the maximum size of duplications retained by all four curators was limited to <500 bp (Tables 1 and 2). We therefore suggest that while one or two curators may be capable of discarding the bulk of obvious putative false positives, adding subsequent curators may increase the putative false-negative rate. We cannot conclude this definitively, as we have not orthogonally verified that the Samplot-rejected SVs were indeed false positives, nor have we orthogonally validated curated SVs. However, because several previous studies have validated Samplot images representing putative true positive variants using e.g. ddPCR or long-read sequencing (Bertolotti et al. 2020; Belyeu et al. 2021), manual curation in Samplot/PlotCritic has in itself been considered an independent validation method to estimate false discovery rates, even without a truth callset (Belyeu et al. 2021; Wold et al. 2023). We therefore refer to both lenient (single curator) and stringent (multiple curator) curated callsets as “high-confidence” SV callsets, sensu Bertolotti et al. (2020).

### Population Structure Is Captured by Curated SVs, But Not by Rejected SVs

As further supporting evidence that high-confidence SV callsets included substantially fewer putative false positives, we compared population structure between curated SVs, Duphold-rejected SVs, curator-rejected SVs, all single nucleotide polymorphisms (SNPs), and short indels as well as downsampled SNPs (to same number as SVs). Assuming that large SVs and SNPs are largely governed by the same evolutionary forces of genetic drift, mutation, recombination, and selection (Lynch 2007; Sjödin and Jakobsson 2012), we hypothesized that even relatively low numbers of common, high-confidence SVs should capture similar patterns of population structure as SNPs and short indels, while SVs rejected as putative false positives would not. In line with our expectations, high-confidence deletions retained by all four curators (Fig. 3a; supplementary fig. S7, Supplementary Material online) best captured population structure (Fig. 3c) inferred from ∼30 million SNPs (Fig. 3b) and 600,575 short indels (supplementary fig. S8A, Supplementary Material online) compared to downsampled SNPs (Fig. 3d; supplementary fig. S8B, Supplementary Material online). In contrast, Duphold-rejected deletions (Fig. 3e) and curator-rejected deletions (Fig. 3f; supplementary fig. S13A, Supplementary Material online), duplications (supplementary fig. S13B, Supplementary Material online), and inversions (supplementary fig. S13C, Supplementary Material online) all largely failed to recover expected patterns. Similar to Bertolotti et al. (2020), we also found that high-confidence deletions best recaptured known population structure from SNPs and short indels compared to duplications and inversions, possibly due to the much lower number of variants remaining after curation (supplementary fig. S7, Supplementary Material online; Table 1).

To further investigate the potential effect of filtering and manual curation on population structure, we calculated pairwise-weighted *F*_ST_ (supplementary table S3, Supplementary Material online) between two individuals for each of four major population clusters (“Trøndelag,” “Pasvik,” “Finland,” and “Leka/Vega”) for the “high-confidence” SV callset retained by all four curators together. We found that relative *F*_ST_ differentiation closely mirrored the relative distance between the major clusters previously identified in the different principal component analyses (PCAs) shown in Fig. 3. Of all SNP and curated SV or rejected SV callsets shown in Fig. 3, the highest weighted *F*_ST_ values were for all pairwise population comparisons for the curated deletion (1,243) callset (Fig. 3a), with strongest differentiation between “Pasvik” and “Leka/Vega” (mean *F*_ST_ = 0.159; weighted *F*_ST_ = 0.275; supplementary table S3A, Supplementary Material online) and weakest differentiation between “Trøndelag” and “Finland” (mean *F*_ST_ = 0; weighted *F*_ST_ = 0.040; supplementary table S3A, Supplementary Material online). Similar to PCA analyses, the callset for Duphold-rejected deletions (1,135) failed to recover any pattern of differentiation (supplementary table S3C, Supplementary Material online).

### Annotation of High-Confidence SVs in *P. domesticus*

To examine the potential impact of SVs, we intersected the high-confidence SV callset with the published annotation for the reference assembly (NCBI accession GCA_001700915.1). High-confidence inversions at least partially overlapped 406 genes, while high-confidence deletions and duplications overlapped 1,277 and 2,570 genes, respectively. As the largest linkage groups, chromosomes 1-5 and 1A and the Z chromosome exhibited the highest numbers of SVs, with the exception that no inversions were retained on the Z chromosome, following curation by a single curator (2,921 SVs; supplementary fig. S4, Supplementary Material online). Notably, five high-confidence duplications (all 20 kb to 2.1 Mb in size) were identified on chromosome 20 (supplementary fig. S16, Supplementary Material online), following both Duphold filtering and manual visual curation. Fifty annotated genes were found to be completely overlapped by a single 1.4 Mb duplication (positions 8119985 to 9530772). All 50 overlapped genes were located with positions 8.16 to 9.46 Mb, ∼841 kb upstream of the *Fm* region, a complex segmental duplication identified on chromosome 20 in the domestic chicken (*Gallus gallus*) genome (Dorshorst et al. 2011; Dharmayanthi et al. 2017). This potential true positive duplication was detected (as heterozygote or homozygote alternate allele) in over 5% of individuals and passed both Duphold (call quality) filtering and a lenient curation strategy. However, it was rejected by multiple manual curators (stringent strategy), highlighting the potential tradeoff between lenient versus stringent curation strategies (supplementary fig. S16, Supplementary Material online; Fig. 4). Given that all short-read SV callsets should be considered preliminary (Wold et al. 2021), further validation with long-read and/or molecular data will be necessary to confirm putative true positive SVs.

We further annotated the 2,921 large, high-confidence SVs retained by a single curator with SnpEff (Cingolani et al. 2012). Of 2,457 deletions, 2% were predicted to be of high impact and 98% were modifiers, while 77%, 9%, and 6% were located within 5 kb of a protein-coding gene in intergenic, intronic, and upstream regions, respectively (supplementary fig. S6, Supplementary Material online). Of 177 inversions, 1% were predicted to be of high impact and 99% were modifiers, while 75%, 13%, 6%, and 5% were located within 5 kb of a protein-coding gene in intergenic, intronic, upstream, and downstream regions, respectively. In contrast, of 287 duplications, 5% were predicted to be of high impact, 38% of moderate impact, and 41% were modifiers, while 41%, 6%, and 29% were located within 5 kb of a protein-coding gene in intergenic, intronic, and transcript regions, respectively.

High-confidence SV callsets were also intersected with a newly generated transposable element library for *P. domesticus* (supplementary figs. S16 and S17, supplementary table S6, Supplementary Material online), using BEDtools v2.29.2 (Quinlan and Hall 2010). High-confidence deletions, duplications, and inversions were all found to overlap mostly with LINE/CR1 and LTR transposable elements identified in the repeat library (see supplementary table S4, Supplementary Material online). Duplications at least partially overlapped with more transposable elements (1,253) compared to inversions and deletions.

## Discussion

The number of population genomic studies on SVs is increasing rapidly. Our aim here was to contribute to best practice in discovering high-confidence SVs, using geographically separated populations of house sparrows in Fennoscandia. In order to do so, we built upon recent insights promoting the use of heuristic-based call/genotype quality filtering (e.g. Liu et al. 2021; Wold et al. 2023) by applying an improved approach for rapid manual curation with Samplot/PlotCritic (Bertolotti et al. 2020; Belyeu et al. 2021). When considering retained high-confidence SVs, we found that these capture similar patterns of population structure to those observed using SNP data. In contrast, SVs rejected by both Duphold filtering (call quality filtering) and manual curation failed to recapture expected population structure (Fig. 3) and differentiation (supplementary table S3, Supplementary Material online) determined with both genome-wide SNPs and curated SV callsets, supporting the conclusion that these rejected variants are indeed likely false positives.

Overall, we note that call/genotype quality filtering alone does not suffice to remove putative false positives. We build upon Wold et al. (2023)'s insights to recommend a time- and cost-effective approach, especially amenable to population genomic and functional genomic projects with resource constraints, e.g. limited to a single reference genome and low-to-medium (<20×) short-read resequencing data. In line with earlier studies (Cameron et al. 2019; Kosugi et al. 2019; Mahmoud et al. 2019; Bertolotti et al. 2020), we found high putative false discovery rates of inferred SVs from short-read data, even when applying a single curator and a lenient manual curation strategy. Our results indicate that future studies could benefit in performing curation/visual inspection of short-read–discovered SVs prior to downstream analyses (Bertolotti et al. 2020), and we recommend different curation strategies based on study objectives below.

### SV Detection in Short-Read Data

Previous population genomic studies reporting SV callsets from short-read data alone have either applied a single program (Catanach et al. 2019; Liu et al. 2021) or a combination of programs (“ensemble algorithms”;, Ho et al. 2020; Weissensteiner et al. 2020), but without heuristic-based filtering (but see Liu et al. 2021; Lee et al. 2023; Wold et al. 2023) and/or manual curation. Where relying on short-read data only, it may ultimately be preferable to apply a single algorithm that uses multiple signals to detect SV presence (e.g. read-depth, split-reads, and read-pairs combined) coupled with quality filtering and manual curation rather than simply overlapping calls from multiple algorithms (Wold et al. 2021, 2023). For example, Cameron et al. (2019) found that no ensemble algorithm (e.g. Parliament2 or SVtools) consistently outperformed individual callers (e.g. LUMPY, Manta, and Delly). In a recent comparison of SV caller and genotyper performance on short-read data generated for an endangered parrot, Wold et al. (2023) found that among competing individual callers/genotypers, smoove (LUMPY/SVTyper) retained the largest number of high-confidence SVs following filtering for quality and size. Additionally, the authors recommended smoove among the best choice of short-read SV discovery tools, should computational and financial resources be limited—as may be the case for many smaller conservation-oriented projects. However, individual callers/genotypers such as smoove are also known to produce high false-positive rates (even following filtering by quality and size), prompting the creation of rapid visual curation methods (Belyeu et al. 2021).

Our most lenient manual curation strategy rejected 29% of deletions, 77.6% of duplications, and 30% of inversions (Table 2), following filtering by genotype frequency (see Materials and Methods; supplementary table S1, Supplementary Material online). Thus, a large fraction of SV calls filtered by genotype frequency are still likely putative false positives in our study, assuming manual curation with Samplot/PlotCritic is accurate, as supported by prior validation efforts of Samplot images (Bertolotti et al. 2020; Belyeu et al. 2021). Similar manual curation results have been observed in other studies utilizing short-read sequencing data. For example, Bertolotti et al. (2020) reported an overall false discovery rate of 91% for SVs initially called with smoove in Atlantic salmon resequenced to 8.1× coverage. This study also validated a subset of SVs retained after visual curation in Samplot/PlotCritic with long-read sequencing and found a putative true positive rate of 88% for presence/absence and 81% for genotype across all SV classes. This suggests that the application of a manual curation pipeline can dramatically reduce putative false-positive calls.

### Considerations and Recommendations for Rapid Manual Curation

Though filtering by call and genotype quality is a good first step for rejecting likely real false positives (e.g. call quality filtering with Duphold: Fig. 3e; Wold et al. 2023), it currently does not suffice in removing the bulk of obvious false positives (Tables 1 and 2 and Fig. 4; Belyeu et al. 2021). In turn, automated curation with Samplot-ML identified even fewer variants as putative false positives than call quality filtering with Duphold, suggesting further fine-tuning on nonhuman callsets is needed. Therefore, manual curation of short-read–discovered callsets is of particular utility in addressing the current constraints for generating high-confidence SV callsets from short-read data alone.

When performing SV discovery from short-read data aligned to a single reference, our results indicate that some degree of curation is better than none at all. Importantly, almost all (>99% of) SVs rejected as putative false positives by a single curator were also rejected by all four curators, showing that there is near-complete agreement between curators in the identification of the most obvious putative false positives. These rejected putative false positives (Fig. 3f; supplementary table S3E, Supplementary Material online) failed to capture the same degree of population structure as curated deletions (Fig. 3a; supplementary table S3D, Supplementary Material online) or SNPs (Fig. 3b and d; supplementary table S3D, Supplementary Material online). In addition, a single, lenient curator rejected up to 80% of SVs as putative false positives, demonstrating that even a minimal investment in time and effort can aid to reject the most obvious putative false-positive calls and substantially improve callsets for downstream analyses and validation. Therefore, if the goal of a given study were to remove as many obvious putative false-positive SVs while minimizing the incidence of putative false negatives, SVs retained by a single curator following SV call/genotype quality filtering could suffice to be considered as a “high-confidence” callset (sensu Bertolotti et al. 2020).

We found that there may be a trend of “diminishing returns” when adding more than two curators, due to an increasing disagreement between what constitutes a putative false-positive SV, especially when study objectives between curators may differ (e.g. rejecting obvious putative false positives vs. retaining only obvious putative true positives; Figs. 2 and 4). Following the addition of a third or fourth curator (the most stringent curators), the percent agreement as to what constitutes a putative false positive decreases substantially between curators (Fig. 4; supplementary table S2, Supplementary Material online), especially for duplications and inversions. For example, the two most lenient curators agreed on 96% of duplications to be rejected (i.e. the remaining 4% had been retained as putative true positive by one but not both of the curators; Fig. 4; supplementary table S2, Supplementary Material online). In contrast, the three most lenient curators only agreed that 79% of the duplications rejected by the fourth and most stringent curator were putative false positives. Within this study, the first author (G.D.) was identified as the most lenient curator because they rejected the fewest variants as putative false positives, while the most stringent curator (A.B.) rejected the most variants. Therefore, callsets retained by more stringent curation strategies necessarily restricted the total number of retained SVs, when different curator callsets are intersected in order of increasing stringency (as in this study; Fig. 4). In the absence of G.D., the number of SVs retained by our next most lenient curator or by the most stringent curator alone would have been significantly fewer. At the same time, variants rejected as putative false positives by the most stringent curation strategy but retained by the most lenient strategy are not necessarily all real false positives.

Even with established guidelines for training new curators (see Supplementary material), we still observed substantial variation in curation stringency. These differences in curation stringency may in large part reflect subtly different perceived project goals: while the most lenient curator aimed to remove obvious false positives, the most stringent curator retained only the most obvious true positives (i.e. only those conforming strongly to expected search images; see Supplementary material), which resulted in large variation in the final callsets retained by different curators. While it is likely that individual variation between curator stringency cannot be completely avoided, prospective studies could substantially reduce this variation by clearly defining curation goals prior to beginning curation. To achieve consistency, it may be helpful for prospective curators to first agree upon project goals: whether the goal is simply to remove obvious putative false positives (lenient strategy) or to attempt to unambiguously identify putative true positives (stringent strategy) while only allowing for Samplot images represented by individuals with correct genotypes—though this latter requirement may be unrealistic for SV genotypes determined from low- to medium-coverage (<20×) short-read data (Wold et al. 2023). It may also be helpful to practice on model data sets or a subset of the real data set and then discuss with curators whether any differences in curator stringency may be due to differences in perceived project goals.

Depending on study objectives, the tradeoff in sensitivity between stringent and lenient curation strategies can be weighed. For example, lenient curation strategies with one or two curators may help to reduce the risk of discarding less obvious putative true positives, particularly for rarer variants such as large, complex SVs exceeding several kilobases where visual curation is more difficult. In contrast, for functional genomic studies (reviewed in Gudmunds et al. 2022), it may be advisable to take a more stringent approach using multiple curators followed by molecular confirmation before further time-intensive functional work. Regardless of study objectives, we consider the combination of heuristic-based quality filtering recommendations (Wold et al. 2023) with rapid visual curation of SV callsets an important and easy first step before drawing inferences from population genomic analyses on small (mostly >25 bp to 1 kb) SVs in both short-read and long-read genomic data sets.

Furthermore, our results suggest that a stringent curation strategy by multiple manual curators may lead to increased false-negative rates, if taking the intersection of calls between all curators (Figs. 2 and 4). We identified a minimum of 29% of SVs designated as putative false positives by visual curation with a single curator and at least 64% with multiple curators. In contrast, quality filtering removed only ∼5% putative false positives and deep learning removed only 2% putative false positives from raw calls. A single curator thus offers a considerable improvement in accuracy compared with filtering and automated approaches and may suffice, at least if the main objective of visual curation is to discard obvious putative false-positive SV calls. In contrast, the use of multiple curators greatly increases accuracy, but at the potential cost of reduced sensitivity (Figs. 2 and 4).

Notably for deletions however, the number, size range, and population structure recovered in PCAs all showed remarkable consistency between a single curator and all four curators (Tables 1 and 2 and Figs. 3 and 4; supplementary fig. S7, Supplementary Material online). In contrast, much more variation was identified between single and multiple curators for the number and size range of inversions and duplications (Tables 1 and 2 and Fig. 4). A total of 434 SVs were found to at least partially overlap annotated genes and also mostly conformed (supplementary fig. S16, Supplementary Material online) to the relative proportions of unannotated SVs retained by a single lenient curator (supplementary fig. S4, Supplementary Material online), while 2,869 short indels partially overlapping genes did not (supplementary fig. S5, Supplementary Material online). The duplication found to overlap 50 genes on chromosome 20 (by a single curator), which may contribute to interesting functional effects, (supplementary fig. S16, Supplementary Material online) was not retained as a putative true positive by all four curators. While the size distributions of retained deletions were also largely similar between single and multiple curators (Tables 1 and 2; supplementary fig. S10, Supplementary Material online), only four SVs retained as putative true positives by multiple curators exceeded 10,000 bp in size, all of which were duplications identified by a single curator. Therefore, adding too many curators will also likely reduce the maximum size of SVs retained as putative true positives, if the intersection of all curation scores is used as in Bertolotti et al. (2020).

We found that filtering by genotype frequency prior to manual curation substantially (but not completely) reduces the proportion of larger variants further (supplementary table S1, Supplementary Material online), implying that larger SVs called with short-read callers (e.g. smoove) are indeed rare. Our lower-than-recommended (Wold et al. 2023) sequencing coverage (∼10×) also likely contributes to the relative paucity of larger variants retained, which (to a certain extent) can be detected by a smoove/Samplot approach (Belyeu et al. 2021). In contrast, in directly applying the same filtering and rapid curation approach (as described here) on higher-coverage (∼30×) short-read data, Smeds et al. (2024) succeeded in detecting and retaining larger high-confidence deletions, duplications, and inversions exceeding 100 kb in size—despite overall putative false-positive rates similar to this study. Regardless, comprehensive resolution of large and complex SVs will require multiple sequencing technologies (high-accuracy long reads and short reads) as well as novel bioinformatic approaches such as pangenome graphs (Sirén et al. 2021). In the absence of these approaches, careful curation of SV calls based on read mapping–based programs alone can aid in narrowing down the list of putative true positive SVs, which may be of biological interest.

To further aid rapid manual curation, we recommend first filtering by genotype frequency, in order to select and plot at least one to three individuals of each genotype. While this restricts SV discovery to only very common variants, our plotting approach is fully scalable to hundreds of samples (Bertolotti et al. 2020), which would increase the potential for detecting relatively rarer variants represented by one to three individuals of each genotype. In testing our approach, we did not find any significant disadvantage to SV curation when reducing the number of individuals of each genotype plotted. Rather, the key benefit appears to be able to contrast individuals of the three genotype classes (homozygote reference, heterozygote, and homozygote alternate) in a consistent order, regardless of whether one or more individuals are visualized per genotype. Recently, this curation approach using only two individuals per genotype class was successfully applied to a study of structural variation across 212 Scandinavian wolves (Smeds et al. 2024), allowing for identification of high-confidence SVs at lower allele frequencies (minor allele frequency [MAF] ≥ 0.01). Crucially, in performing manual curation with Samplot/PlotCritic, Smeds et al. (2024) were able to remove batch effects discernible in the raw SV calls and rejected calls also failed to recapture expected population structure (as found in this study).

We have here defined a putative false-positive variant as an SV call not passing call/genotype quality filtering or manual curation. Supporting this assumption, rejected variants failed to recapture expected population structure (Fig. 3d and e; supplementary table S3C and E, Supplementary Material online). We note again, however, that we have not applied orthogonal evidence to verify that calls identified as putative false positives are not e.g. true positive calls with relatively “poor” concordant/discordant read-pair and split-read signals in Samplot. In theory, it may be possible that even a relatively unambiguous putative false positive (e.g. rejected unanimously by all four curators) could indicate the presence of a true complex variant, which is difficult to resolve with short-read data. However, in line with previous studies (Bertolotti et al. 2020; Belyeu et al. 2021), we suspect that variants identified as false positives in Samplot harbor a disproportionately higher probability of being erroneous, especially when discovered by aligning short reads to older (e.g. Illumina) reference genomes. Other than high error rates previously documented for SV discovery tools and short-read data themselves (e.g. due to alignment artifacts), possible sources for erroneous calls could include gaps or misassemblies in the reference genome, library preparation, polymerase chain reaction (PCR) artifacts, or somatic SVs (Cameron et al. 2019; Mahmoud et al. 2019). However, even if putative false positives were to indirectly point toward the presence of a large/complex SV, the number, size, and orientation of the smaller calls would likely still be erroneous. If numerous, these erroneous calls could substantially inflate allele counts and downstream population genetic summary statistics (see below). We therefore distinguish here between indirect evidence (e.g. through erroneous mapping signals) for the possible presence of a novel and complex SV versus direct characterization of the number of (mostly smaller) putative true positive SVs of specific size and orientation reflecting actual biological differences in genomic architecture across individuals.

Ultimately, it may be impossible to completely avoid the tradeoff between removing putative false positives at the cost of removing putative true positives with manual visual curation alone. This tradeoff has however nearly been achieved in human genomic studies (Belyeu et al. 2021) with call quality (Duphold) filtering and automated curation of deletions using deep learning (Samplot-ML), though these tools remain optimized for human data. Continued development and refinement of automated deep learning methods for reliable curation of a broader range of SV classes in both short- and long-read data will prove to be of particular utility for future genomic studies on wild organisms.

### Implications for Downstream Population Genetic Analyses of SVs

Most population genetic studies of SVs do not perform broad-scale visual curation on their SV callsets (Bruders et al. 2020; Catanach et al. 2019; Dorant et al. 2020; Liu et al. 2021; Rinker et al. 2019; Weissensteiner et al. 2020; Wold et al 2023). However, both our study and previous studies (Bertolotti et al. 2020; Belyeu et al. 2021) have shown that rapid visual curation of SVs can easily detect and remove a large number of putative false positives. Even with the approach presented here (which excludes rare variants), provided larger sample sizes, prospective studies can easily inspect SV calls with allele frequencies at a standard MAF of 5% or less (Figs. 1 and 2). Importantly, we found that larger SVs (>5 kb) were both called at lower frequencies (supplementary table S1, Supplementary Material online) and prone to higher putative false-positive rates (Table 2). In particular, the presence of false-positive SV calls at lower frequencies (especially those called/genotyped at <20× coverage) could inflate the relative proportion of lower-frequency variants in e.g. site frequency spectra-based analyses. Therefore, studies inferring population genetic statistics from uncurated SV callsets may be biased by high false-positives rates.

Before we can gain a detailed understanding of the population genetic nature of SVs, a combination of both assemblies generated from high-accuracy long-read data (in the form of a pangenome graph) and population-level short-read resequencing data will be needed to expand the known range of SV variation in populations (Sirén et al. 2021; Wold et al. 2021; Liao et al. 2023; Shi et al. 2023). In particular, large SVs that vastly exceed the insert size range as well as those in highly repetitive regions are inherently harder to detect by both short-read mapping tools and manual visual curation. However, future developments could improve rare SV detection in wild populations, by leveraging manually curated SV callsets as training data for artificial intelligence-based detection methods (Cleal and Baird 2022), as well as for use with other high-confidence callsets when constructing pangenome variant graphs (Sirén et al. 2021; Liao et al. 2023).

## Conclusion

As important determinants of both deleterious and adaptive phenotypic effects, SVs are increasingly targeted for evolutionary genomic studies of wild populations, including those of conservation concern. Such studies may often be constrained by computational resources or time, as well as funds to adequately resequence individuals to recommended coverage thresholds for short-read SV calling/genotyping (e.g. >20× to mitigate false discovery rates and genotyping errors; Wold et al. 2023) or to orthogonally validate putative true positive variants. We outline an easy and cost-effective strategy for enriching low-to-medium–coverage short-read SV callsets with high-confidence variants, using only a single reference assembly. In complementing heuristic-based quality filtering with rapid manual curation in a wild animal species, we demonstrate the feasibility of this approach in forming a part of short-read SV detection pipelines. Prior to curation, the permissible putative false-positive and false-negative rates (lenient vs. stringent curation strategies) may be chosen according to project goals (Fig. 2) and prospective curators trained accordingly. For example, a single curator applying a lenient curation strategy may suffice for population genomic studies attempting to characterize a broader pool of high-confidence SVs, while a stringent strategy applied by multiple curators may be necessary for functional validation studies or selecting probes for array design. Given that a high-confidence SV catalog generated from multiple long-read assemblies (e.g. a pangenome) will still be lacking for most genomic studies on wild populations, time- and cost-effective filtering and curation of short-read–discovered SVs present an important alternative.

## Materials and Methods

### Sampling and Sequencing

DNA was collected from 33 house sparrow individuals from locations in Norway and Finland (see fig. S1 and table S2 in Lundregan et al. (2018), for map and details of sampling sites, respectively). Blood samples were taken from the brachial vein, and DNA was extracted as described in Hagen et al. (2013) using the ReliaPrep Large Volume HT gDNA Isolation System (Promega) automated on a Biomek NXp pipetting robot (Beckman Coulter). Samples were sequenced using a 100-bp paired-end Illumina TruSeq protocol with a short insert size library of ∼180 bp on 21 lanes on the HiSeq 2000 platform to a targeted average depth of ∼10× (Elgvin et al. 2017). Adapter sequences were trimmed from raw reads using cutadapt v.2.3 (Martin 2011) with options “--minimum-length = 30 --pair-filter = any”. An overview of the number of reads for each sample before and after filtering is provided in supplementary table S5, Supplementary Material online.

### Short Indel Calling

Trimmed reads were aligned with BWA-MEM (bwa v.0.7.17) to the short-read reference genome assembly for *P. domesticus* (Elgvin et al. 2017; GCA_001700915.1_Passer_domesticus-1.0), available at https://www.ncbi.nlm.nih.gov/assembly/GCA_001700915.1/ and then sorted and indexed with Samtools v.1.9. All unplaced scaffolds were removed, and thus only scaffolds mapped to chromosomal regions were included in downstream analyses. Short indels were called and genotyped with GATK v.4.1.4.1 using the HaplotypeCaller and GenotypeGVCFs functions. Short indels were extracted from the resulting joint-called .vcf file with “SelectVariants -select-type INDEL” and then filtered using “VariantFiltration” with the recommended filter expressions from GATK (McKenna et al. 2010) using “QD < 2.0”, “QUAL < 30.0”, “FS > 200.0”, and “ReadPosRankSum < - 20.0”.

### Large SV Calling and Genotyping

Among generalist SV callers for low-to-medium–coverage short-read data sets, LUMPY performs with higher sensitivity compared to other common programs (Cameron et al. 2019). We therefore called larger (>20 bp) SVs (deletions, duplications, and inversions) from the aligned .bam files using LUMPY (Layer et al. 2014) and genotyped the resulting calls with SVTyper (Chiang et al. 2015), via the smoove pipeline (Pedersen et al. 2020). This resulted in a file of genotyped SVs (homozygote reference, heterozygote or homozygote alternate) that was then queried with BCFtools (Danecek et al. 2021) for polymorphic SV calls with “bcftools query -f ‘%CHROM\t%POS\t%END\t%ALT[\t%GT]\n’” (Li et al. 2009).

### Heuristic-Based Call and Genotype Filtering with Duphold and MSHQ

As per recommendations from Wold et al. (2023) and Pedersen and Quinlan (2019), we filtered raw callsets for call quality and genotype quality. We filtered deletions and duplications with Duphold (Pedersen and Quinlan 2019), a heuristic-based filtering tool that excludes suspected false positives based on fold-change thresholds applied to regions 1 kb in length adjacent to putative SVs. We applied DHFFC thresholds by only retaining putative deletions with “DHFFC < 0.7” and duplications with “DHFFC > 1.3” in BCFtools. We additionally filtered all SV classes (deletions, duplications, and inversions) with Mean Smoove Heterozygote Quality scores provided by smoove, retaining only variants with a genotype quality score above “MSHQ > = 3” (alternate variants with heterozygote individuals) or equal to “MSHQ = -1” (alternate variants with homozygote individuals only) in BCFtools.

### Automated Filtering with Samplot-ML

A deep learning approach using Samplot-ML (Belyeu et al. 2021) was used to curate putative deletions with a convolutional neural network algorithm adapted to Samplot. Samplot-ML is not yet available for automated curation of duplications and inversions.

### Visual Curation with Samplot/PlotCritic

Samplot (Belyeu et al. 2021) was used to generate .png files to visualize SVs for manual visual curation (see Supplementary material). A custom Python script (gen_samplot.py) was used to select SVs represented by at least three individuals of the homozygote reference, homozygote alternate, and heterozygotes (see Fig. 1) and to generate plots of least two to three individuals per genotype. Occasionally, one to two individuals were repeated in the same Samplot due to sampling with replacement, though the overall effect on curation was deemed to be at most negligible for the focus of this study. Juxtaposition of individuals from each of the three genotype classes increased both speed and accuracy during SV call curation. PlotCritic websites were established separately for each variant class (deletion, duplication, or inversion) via an Amazon Web Services Instance, using commands provided through SV-Plaudit pipeline (Belyeu et al. 2018; note: PlotCritic is now available independent of AWS; Belyeu et al. 2021). All four curators were provided guidelines for identifying putative true positive SVs, from features previously agreed upon by G.D. and A.B (see “Appendix” in Supplementary Material online). In order to contrast different curation strategies (e.g. lenient: remove obvious putative false positives; stringent: identify unambiguous putative true positives with only correct genotypes), each SV was then given a score of “Yes,” “Maybe,” or “No” during visual inspection by curators G.D., H.A.B., and E.G., but only “Yes” or “No” by A.B.

Summary reports were downloaded from each PlotCritic website, and curation scores were extracted. For each variant class, a .bed file of all variants receiving the score “Yes” was created for each curator. To create the final curated set of high-quality SVs, the .bed files across all four curators were intersected using BEDOPS v. 2.4.39 with the --intersect flag (Neph et al. 2012). The resulting .bed file was then intersected with the bcftools query .bed file for all SVs generated earlier, by specifying 90% reciprocal overlap “-f 0.9 -r” with BEDTools v2.29.2 (Quinlan and Hall 2010) to filter out SVs with largely redundant overlap. In this study, we define “putative true positive” (high-confidence) SVs as confirmed by one or more curators, while “putative false positive” SVs were rejected by one or more curators (Pedersen and Quinlan 2019). We note however that we have not functionally validated the SVs (see Discussion).

### SV Annotation and Functional Effect Prediction

Curated SVs were annotated and their functional effect predicted using SnpEff v. 4.3t, with the putative impact defined as “low,” “medium,” or high” according to Cingolani et al. (2012). Partial and complete (100%) overlap of annotated genic regions (using available annotation for the GCA_001700915.1 genome; Elgvin et al. 2017) with curated SVs was determined with BEDTools v2.29.2 (Quinlan and Hall 2010). Size distributions were calculated from the curated SV bed files using the Pandas library (McKinney 2012) in Python3 (Van Rossum and Drake 2009). Curated SVs were also intersected with a repeat library (see below), using BEDTools v2.29.2 (Quinlan and Hall 2010).

### Repeat Library Construction

Repetitive elements were identified using the Earl Grey TE annotation pipeline (version 1.2; Baril et al. 2021, 2022), configured with Repbase (version 23.08) and Dfam (version 3.4) repeat libraries (Jurka et al. 2005; Hubley et al. 2016). Briefly, Earl Grey first annotated known repeats using the *Aves* repeat library. Following this, Earl Grey identified and refined novel TEs using an automated and iterative implementation of the “BLAST, Extract, Extend” process (Platt et al. 2016). Following final TE annotation, overlapping and fragmented annotations were resolved by Earl Grey before final TE quantification.

### Population Structure Analyses

We compared the PCA using SVs, with the PCA of all genotype likelihoods for SNPs estimated with the GATK model “-GL 2, -doGlf 2 -SNP_pval 1e-6, -doMajorMinor 1 -doMaf 2 -minMapQ 30 -minQ 20” with ANGSD v. 0.921 (Korneliussen et al. 2014). Covariance matrices for the genotype likelihoods of SNP and SV callsets were extracted using PCAngsd (Meisner and Albrechtsen 2018), decomposed in R (R Core Team 2020), with scripts from Mérot et al. (2023) and plotted with Python3 (Pedregosa et al. 2011). We then compared population structure recaptured with SVs both retained and rejected by curators to 1,200 and 15,000 randomly downsampled SNPs obtained from ANGSD with “-doGeno 4, -doPlink 2”. Downsampling was performed using PLINK v1.90 (Purcell et al. 2007) with “--thin-count” flag, with the subset of downsampled SNPs roughly equal to the number of loci in our callsets of the four curator callsets (1,243 deletions) and uncurated deletions (15,029).

Pairwise mean and weighted *F*_ST_ (as defined by Weir and Cockerham 1984) was calculated using the --weir-fst option in VCFtools (version 0.1.16) for the following data sets: all ∼30 × 10^6^ raw SNPs, 1,200 randomly downsampled SNPs, 1,243 high-confidence deletions retained by all curators, 1,004 deletions rejected after curation, 1,135 deletions rejected after filtering with Duphold (DHFFC <0.7), and all raw unfiltered and uncurated deletions. Because a key population (“Pasvik”) was represented by only two samples, we chose two individuals for each of the four major population clusters identified with the PCAs that were chosen for *F*_ST_ comparisons: “Trøndelag”: individuals 8L52141 and 8L52815; “Pasvik”: 8L19747 and 8L19766; “Finland”: FIN33 and FIN248; and “Leka/Vega”: 8L64093 and 8N73248 (see fig. S1 and table S2 in Lundregan et al. (2018), for further sample location details).

## Supplementary Material

evae049_Supplementary_Data

## Data Availability

The Illumina reads and assembled reference genome from this article are available at NCBI, BioProject number PRJNA255814 (*P. domesticus* reference accession number SAMN02929199). Additional data and script are available at the Dryad database: https://datadryad.org/stash/share/lik6XKWSVLN5pyVhIxOkcxrhDAqSH_sFNmEnSdTMwyo.
