# Supplementary material for: Calling Structural Variants with Confidence from Short-Read Data in Wild Bird Populations
Source: Genome Biol Evol. 2024 Mar 15;16(4):evae049. doi: 10.1093/gbe/evae049 (PMC11018544; doi:10.1093/gbe/evae049)
Supplement: evae049_Supplementary_Data [file evae049_supplementary_data.zip › Supplemental _Materials_for_GBE_February_2024.pdf]

Supplemental Materials for:

Calling structural variants with confidence from short-read data in wild bird populations

Gabriel David1*, Alicia Bertolotti2, Ryan Layer3, Douglas Scofield1, Alexander Hayward4, Tobias Baril4, Hamish Burnett5, Erik Gudmunds1, Henrik Jensen5, Arild Husby1*

1 Evolutionary Biology, Department of Ecology and Genetics, Evolutionary Biology Centre, Uppsala University, Uppsala, Sweden.

2 School of Biological Sciences, University of Aberdeen, Tillydrone Avenue, Aberdeen, UK

3 BioFrontiers Institute, University of Colorado, Boulder, CO, USA, Department of Computer Science, University of Colorado, Boulder, CO, USA

4 Centre for Ecology and Conservation, University of Exeter, Penryn Campus, Penryn, Cornwall, TR10 9FE, UK

5 Centre for Biodiversity Dynamics, Department of Biology, Norwegian University of Science and Technology, Trondheim, Norway

Table of Contents:

**Figure S1. Example of the PlotCritic interface, showing the curation question and the set of answer options which when selected are recorded for each individual curator.**	Page 4
**Figure S2. Example PlotCritic visualization, showing 3 individuals for each genotype (homozygote reference, heterozyogte, homozygote alternate) for a putative true positive Deletion.**	Page 5
**Figure S3. Example PlotCritic visualization, showing a putative false positive inversion.**	Page 6
**Figure S4. Number of curated structural variants by linkage group (single curator).**	Page 7
**Figure S5. Distribution of genes partially overlapped by short indels, by linkage group.**	Page 8
**Figure S6. Percentage of high impact annotations by structural variant class.**	Page 9
**Figure S7. Population structure visualised using PCAs with uncurated and curated structural variants callsets, by variant class.**	Page 10
**Figure S8: Population structure shown by PCA generated with (a) 600,575 short indels, compared to that shown by (c) 15,000 downsampled SNPs called with the GATK model in ANGSD and (c) genotype likelihoods called by the GATK model in ANGSD and plotted with PCAngsd.**	Page 11
**Figure S9. Size histograms in total basepairs for uncurated SVs and curated SVs.**	Page 12
**Figure S10. Size distribution of deletions retained above and below 500 bp in single and four-curator callsets.**	Page 13
**Figure S11. Size distribution of duplications retained above and below 500 bp in single and four-curator callsets.**	Page 14
**Figure S12. Size distribution of inversions retained above and below 500 bp in single and four-curator callsets.**	Page 15
**Figure S13. Population structure shown by PCA generated with all rejected structural variants marked as “No” by four curators.**	Page 16
**Figure S14. Proportion of repeat elements in *Passer domesticus* genome, by class.**	Page 17

**Figure S15. Proportion of repeat elements in *Passer domesticus*, by genome compartment.**	Page 18
**Figure S16. Number of genes at least partially overlapped by 434 curated SVs (retained by the most lenient curator) across the different linkage groups for *Passer domesticus*. Figure S19. Number of genes at least partially overlapped by 434 curated SVs (retained by the most lenient curator) across the different linkage groups for *Passer domesticus***	Page 19
**Table S1. % variants removed following filtering by "genotype-frequency", prior to manual curation (SVs selected for curation which are represented by at least three individuals of each genotype class).**	Page 20
**Table S2. Total SV calls, Putative False Positive Rate and % Agreement between curators.**	Page 21
**Table S3. Mean and Weighted FST for population structure comparisons in Figure 3. Weighted FST values are in parentheses.**	Page 22
**Appendix: Identifying Structural Variants with Samplot**	Page 24

Figure S1. Example of the PlotCritic interface, showing the curation question and the set of answer options which when selected are recorded for each individual curator.

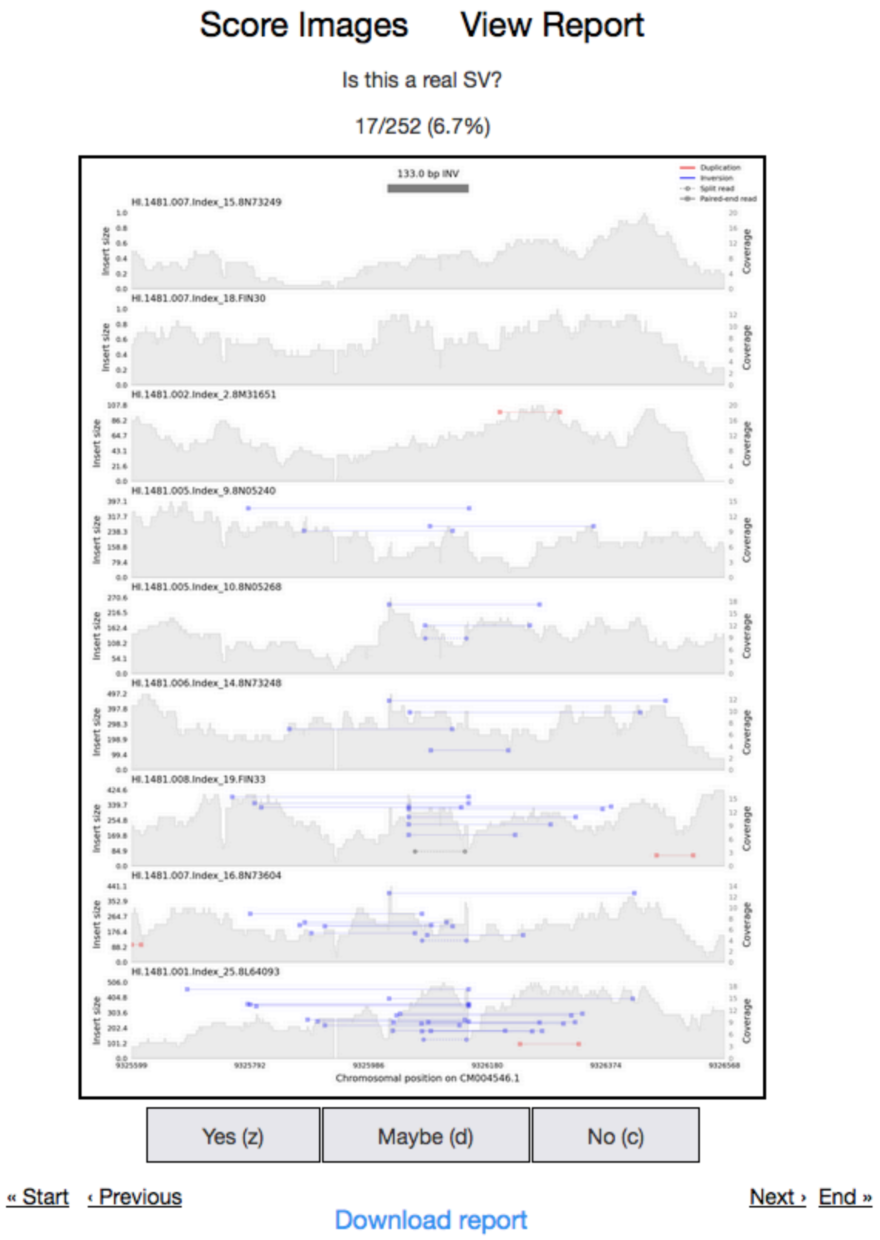

Figure S2. Example of a Samplot visualisation scored in PlotCritic, showing 3 individuals for each genotype (homozygote reference, heterozyogte, homozygote alternate) for a putative true positive Deletion.

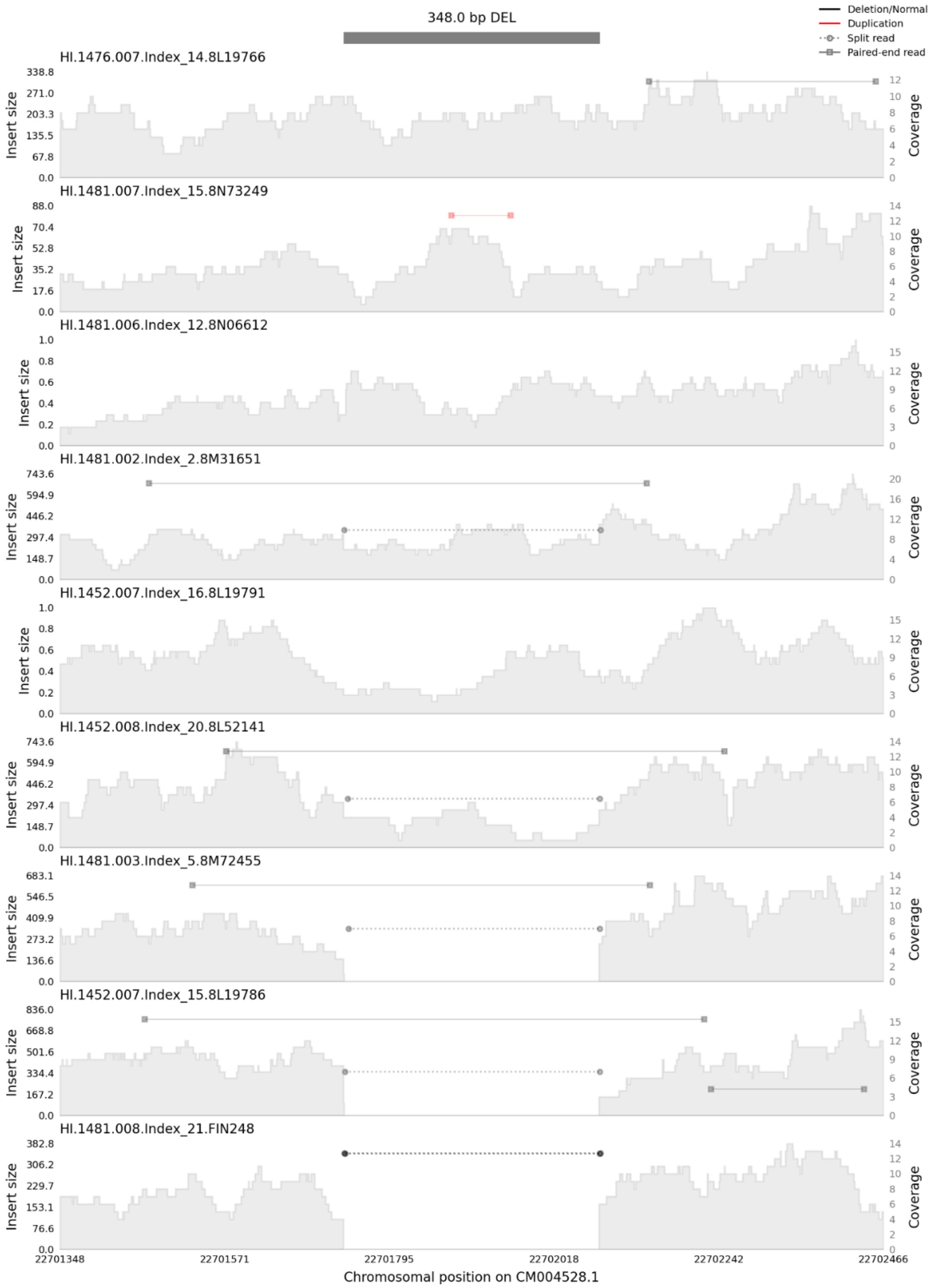

Figure S3. Example PlotCritic visualization, showing a putative false positive inversion.

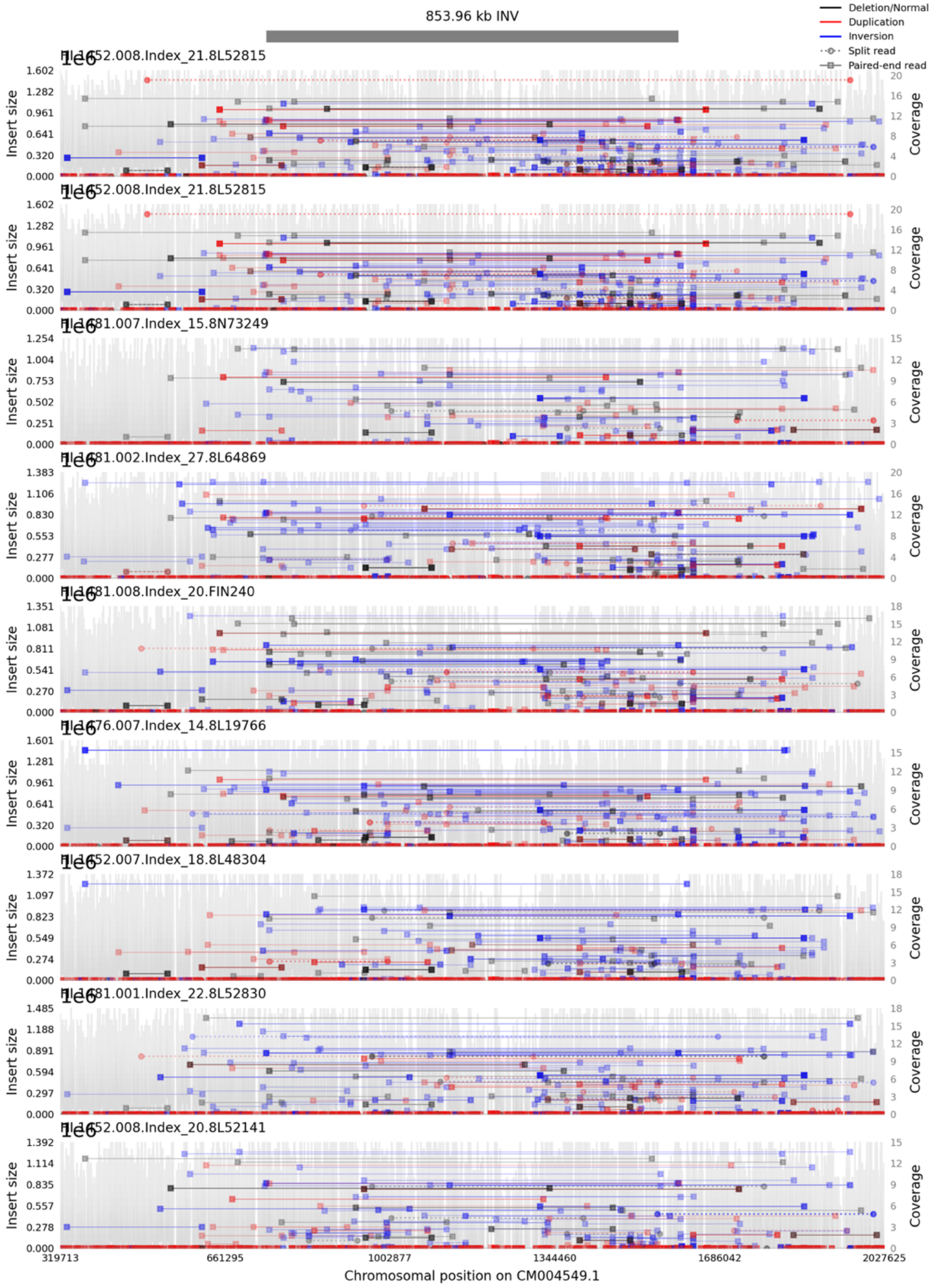

Figure S4. Number of curated structural variants by linkage group (single curator).

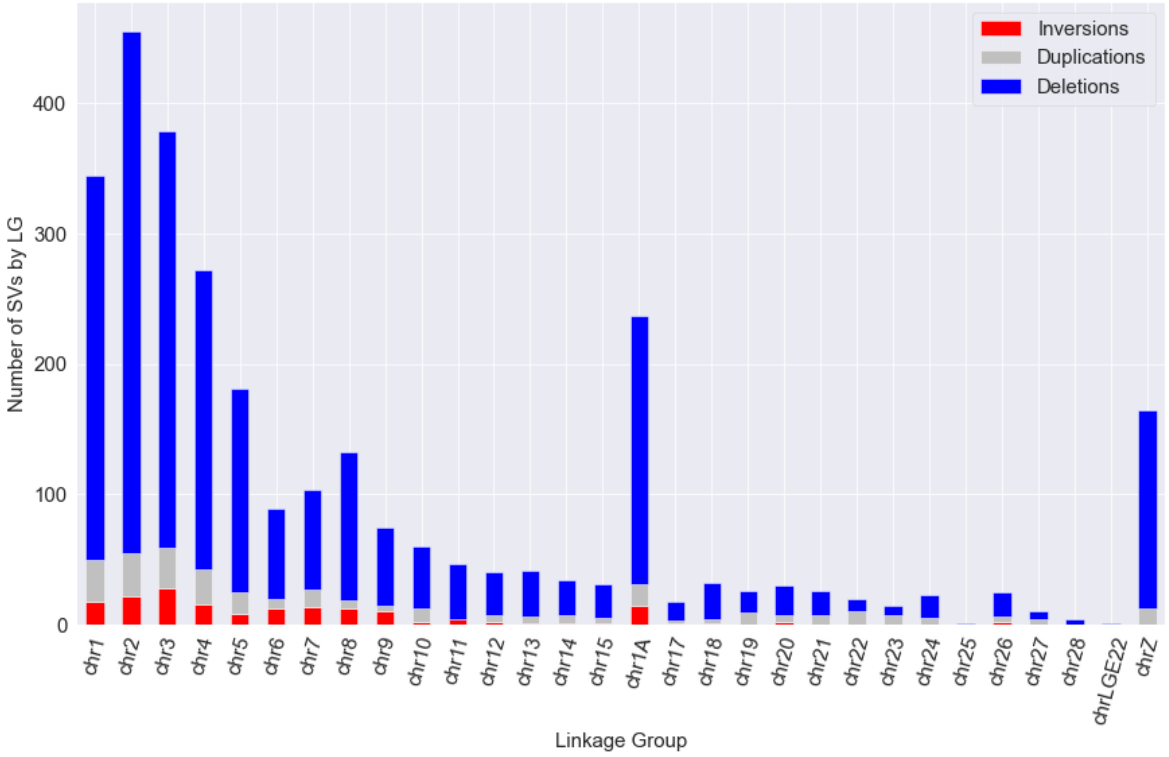

Figure S5. Distribution of genes partially overlapped by short indels, by linkage group.

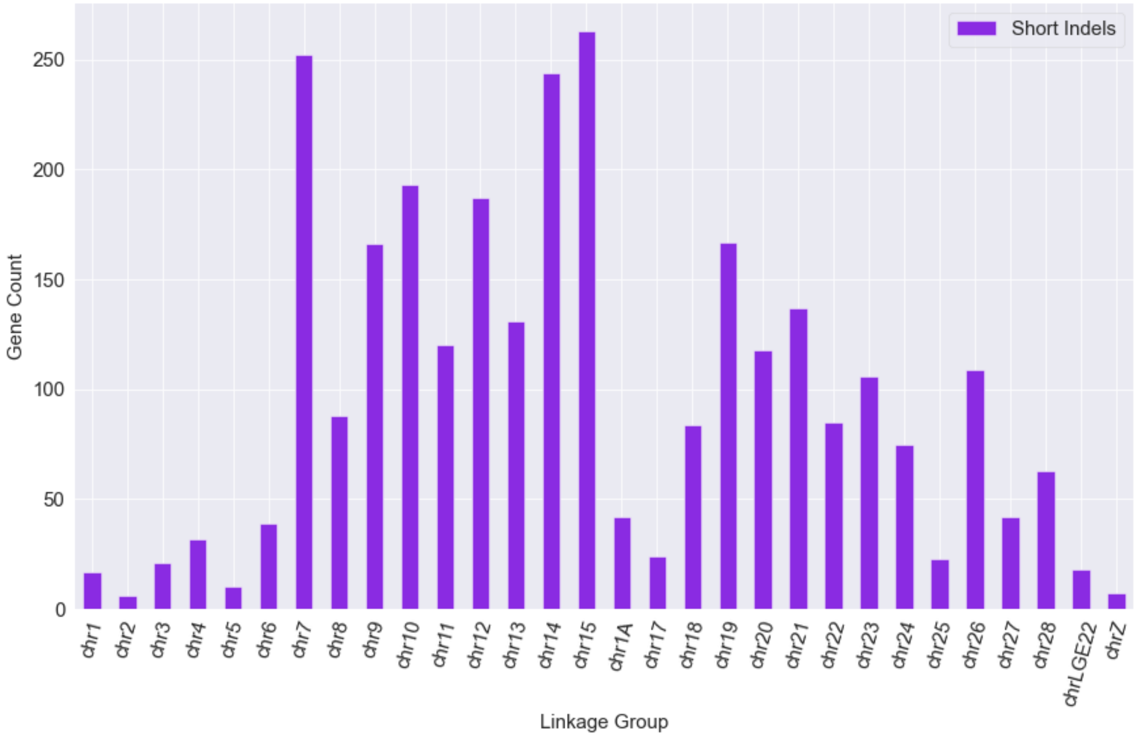

Figure S6. Percentage of high impact annotations by structural variant class.

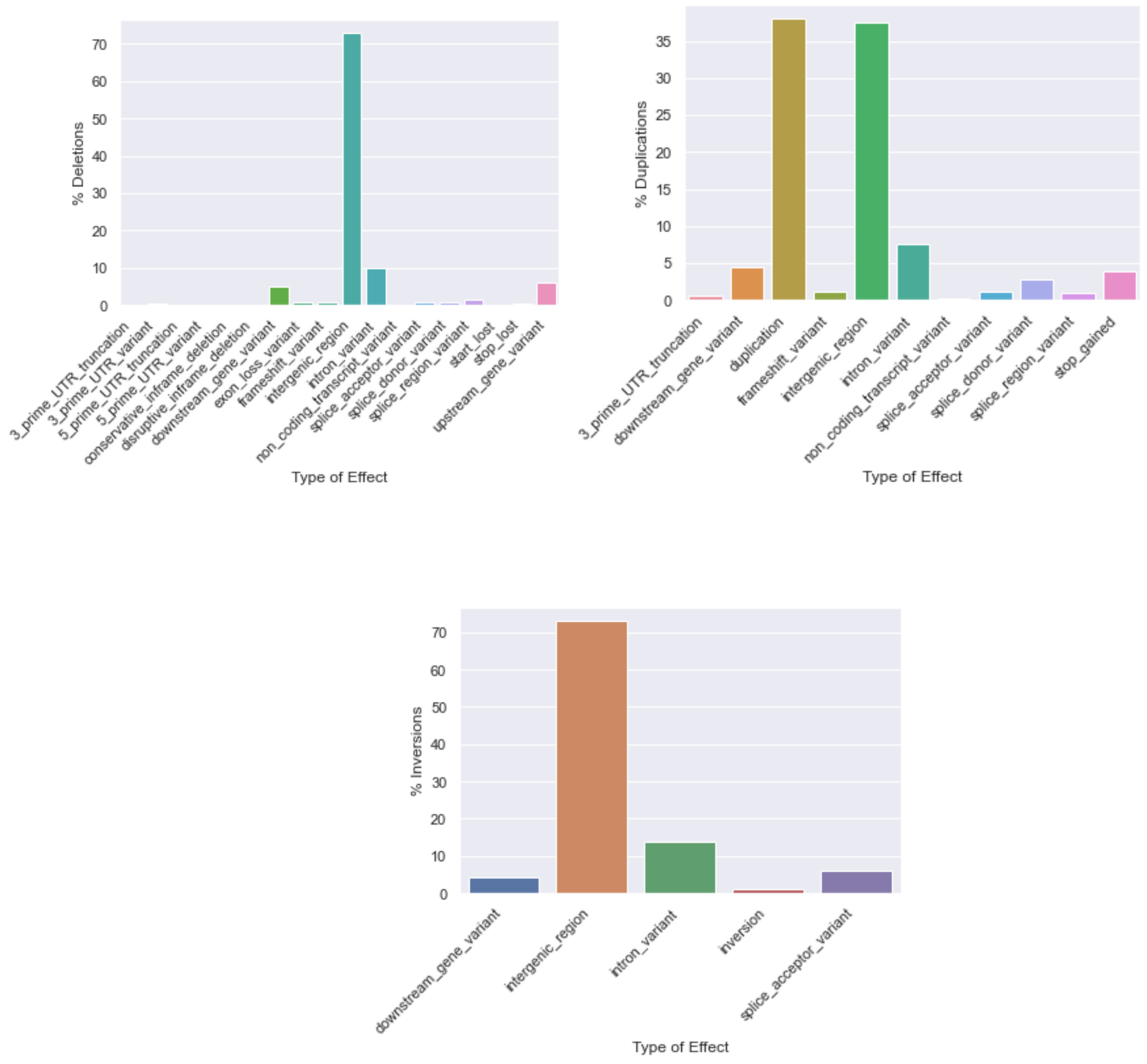

Figure S7. Population structure visualised using PCAs with uncurated and curated structural variants callsets, by variant class. Top row: deletions retained by a single curator (2457), duplications retained by a single curator (287), inversions retained by a single curator (177); Bottom row: deletions retained by four curators (1243), duplications retained by row curators (37), inversions retained by four curators (13). Colours correspond to populations specified in Figure S6. ALTA (grass green)=Alta; HELG (orange)=Helgeland; TRON(light pink)=Trøndelag; PASV (bright pink)=Pasvik; VEGA(army green)=Vega; LEKA(dark purple)=Leka; LEKAxVEGA (light blue)=Leka x Vega; FIN(bright violet)=Finland.

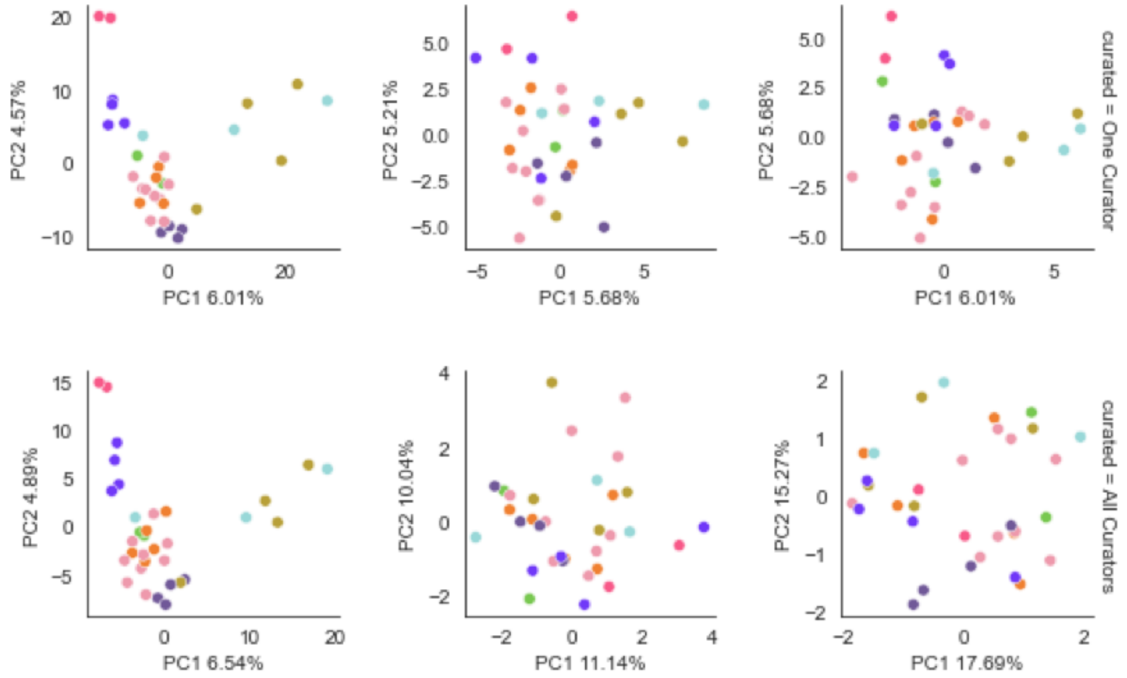

Figure S8: Population structure shown by PCA generated with (a) 600,575 short indels, compared to that shown by (b) 15,000 downsampled SNPs called with the GATK model in ANGSD and (c) genotype likelihoods called by the GATK model in ANGSD and plotted with PCAngsd.

a)

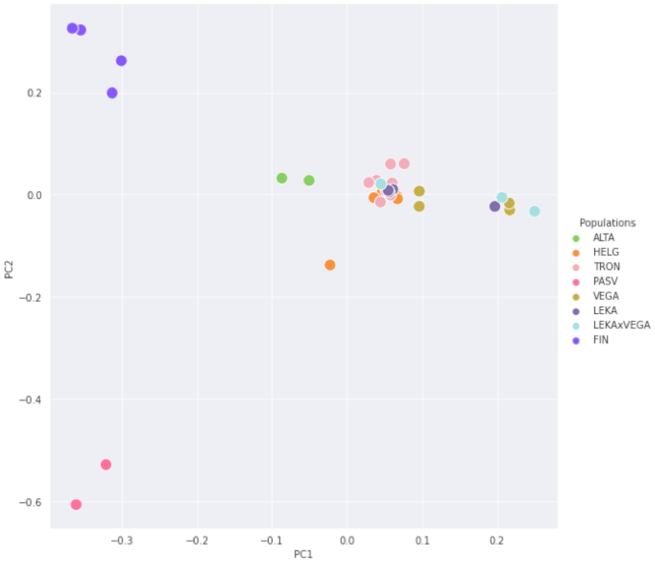

b)

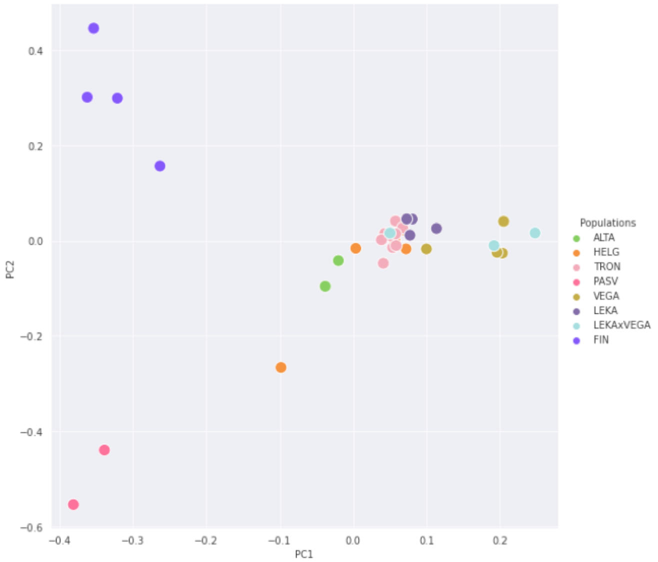

c)

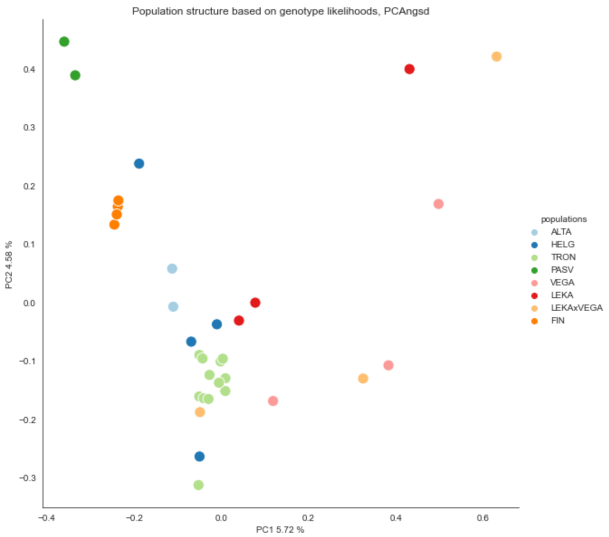

Figure S9. Size histograms in total basepairs for uncurated SVs and curated SVs. Top row: a) uncurated deletions (15,029); b) uncurated duplications (3430); c) uncurated inversions (1188). Middle row: c) curated deletions, single curator (2457); d) curated duplications, single curator (287); e) curated inversions, single curator (177). Bottom row: g) curated deletions, four curators (1243); h) curated duplications, four curators (37); i) curated inversions, four curators (13). An arbitrary maximum cut-off of 5000 bp was applied when plotting. The small peak around 400bp in the uncurated deletions a) coincides with the insert size (~380 bp). Note the different scale on the y-axis in the plots.

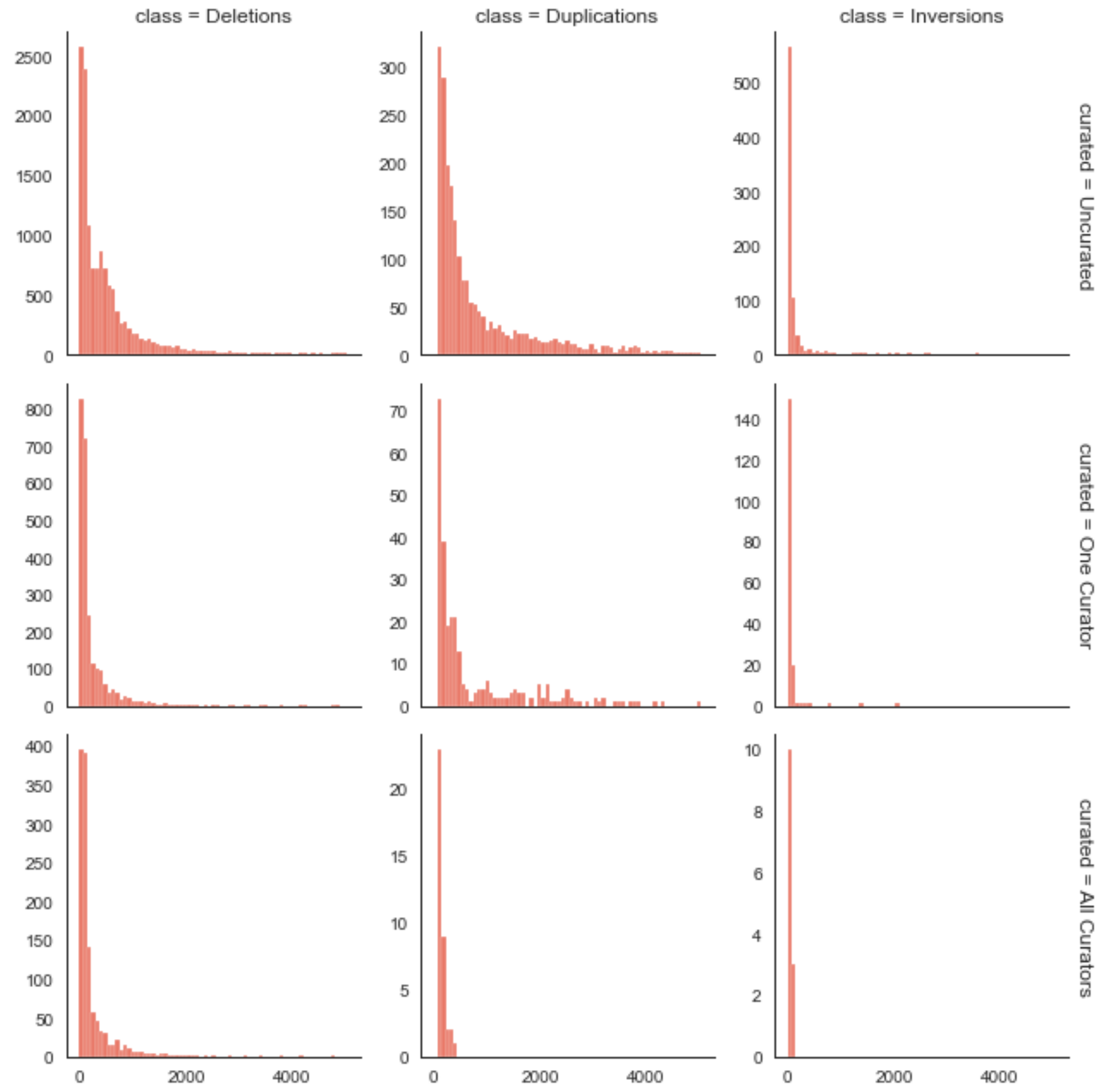

Figure S10: Size distribution of deletions retained above and below 500 bp in single and four-curator callsets.

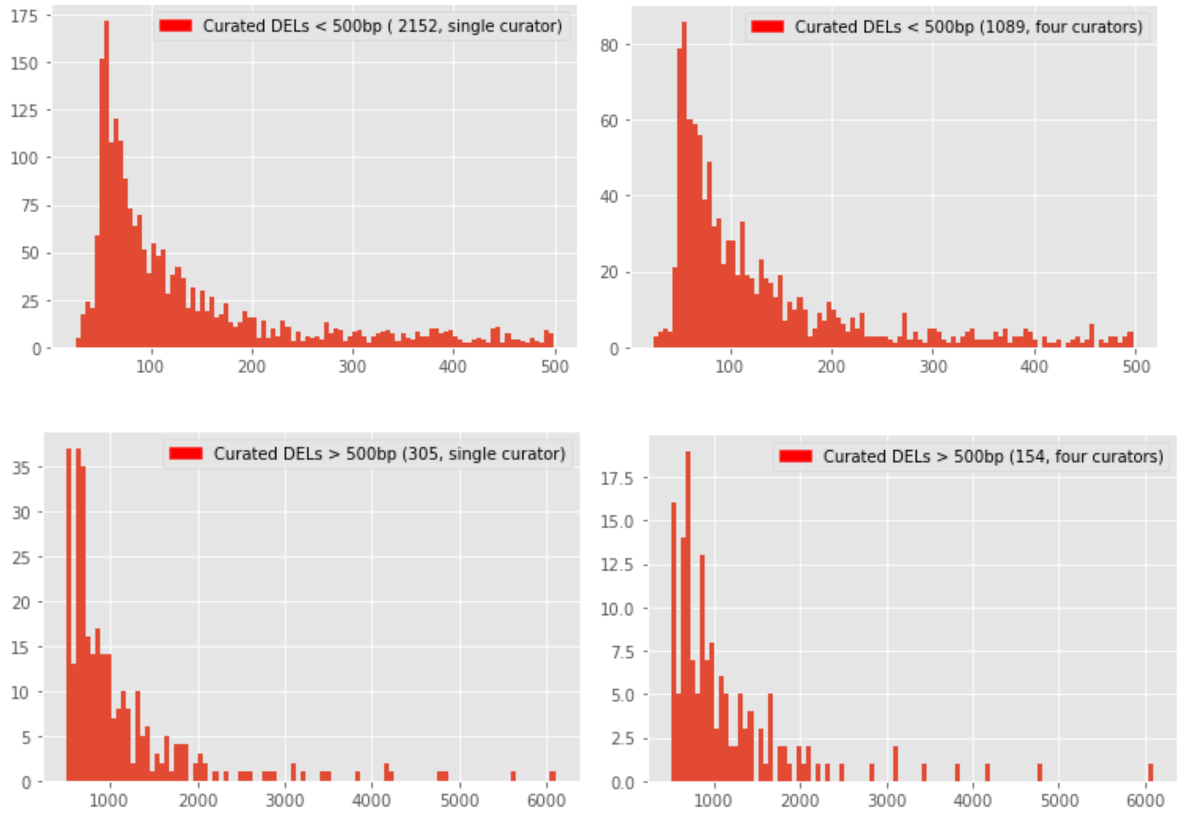

Figure S11. Size distribution of duplications retained above and below 500 bp in single and four-curator callsets.

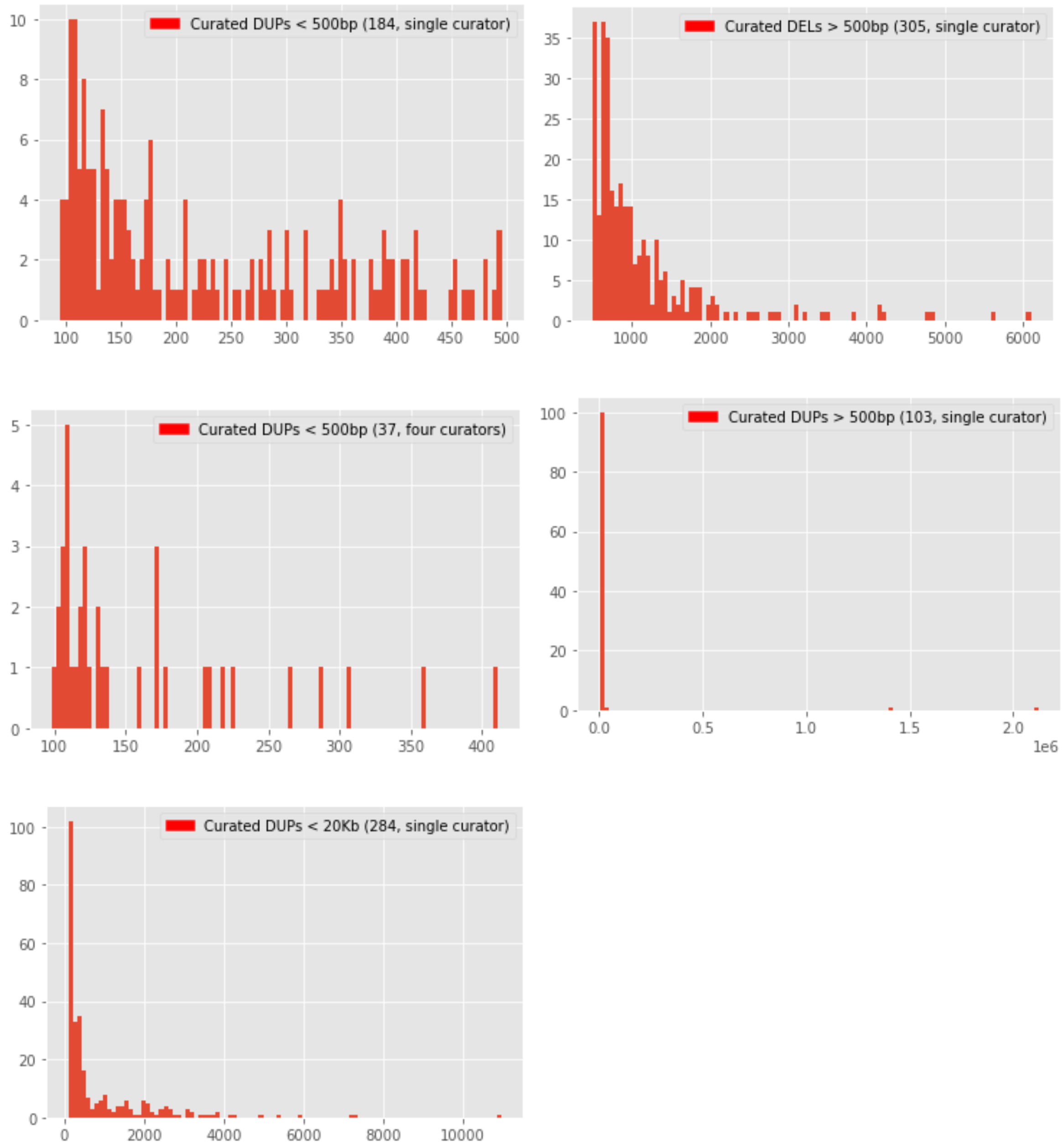

Figure S12. Size distribution of inversions retained above and below 500 bp in single and four-curator callsets.

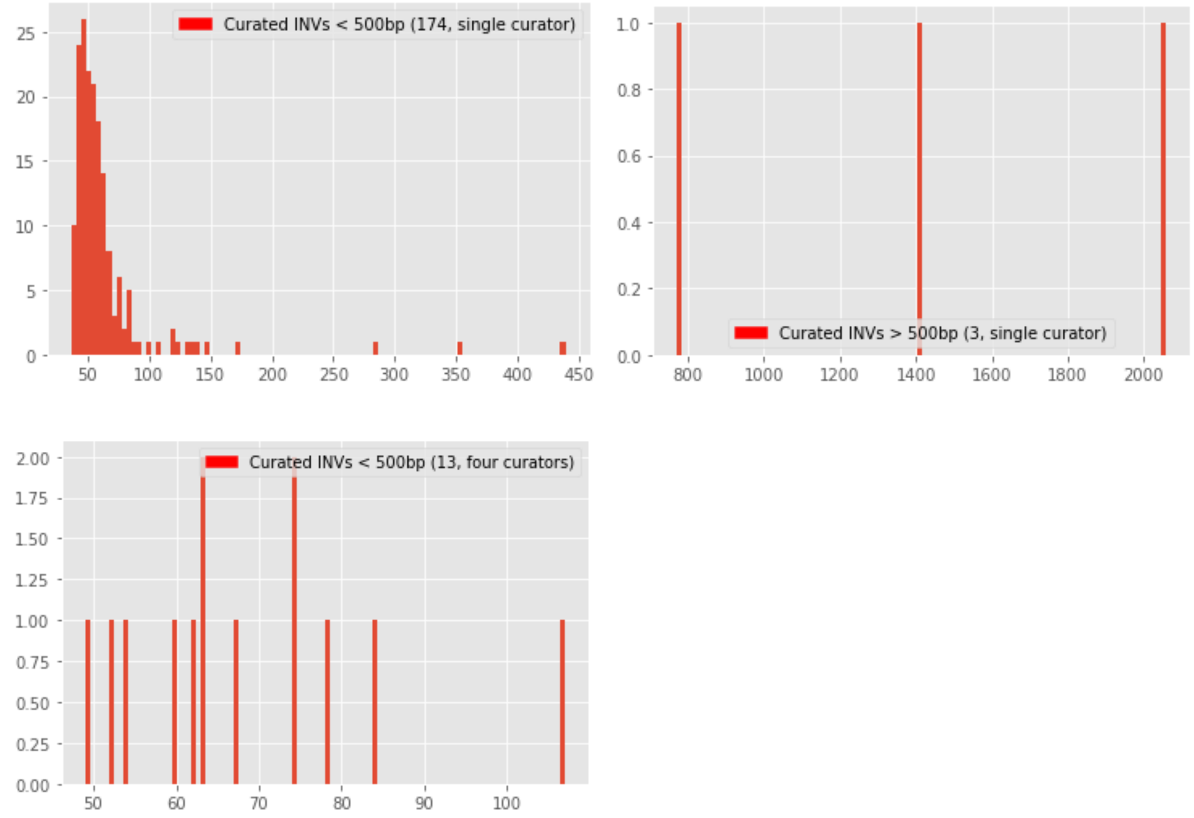

Figure S13. Population structure shown by PCA generated with all rejected structural variants marked as "No" by four curators; a) 729 deletions; b) 552 duplications; c) 51 inversions.

a)

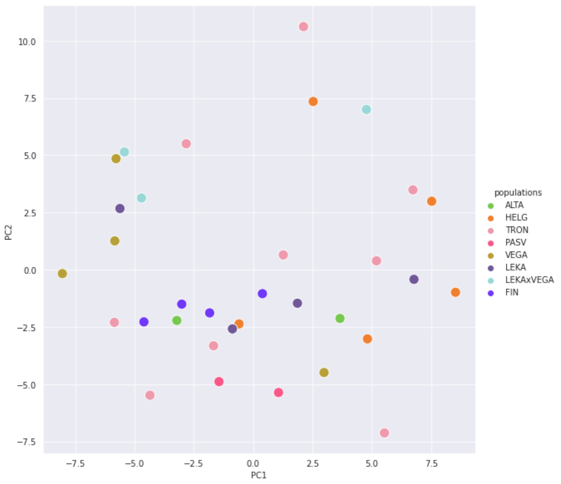

b)

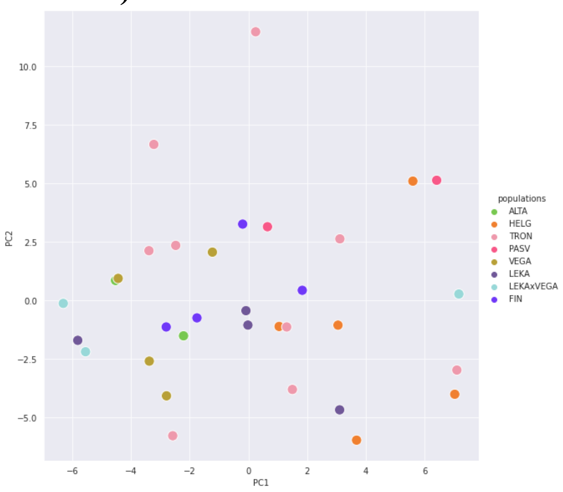

c)

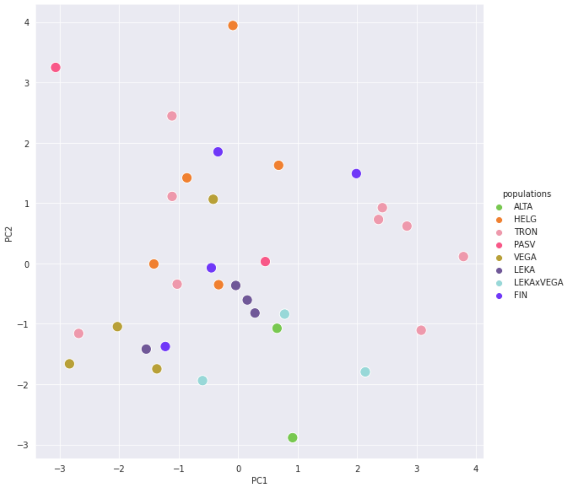

Figure S14. Proportion of repeat elements in *Passer domesticus* genome, by class.

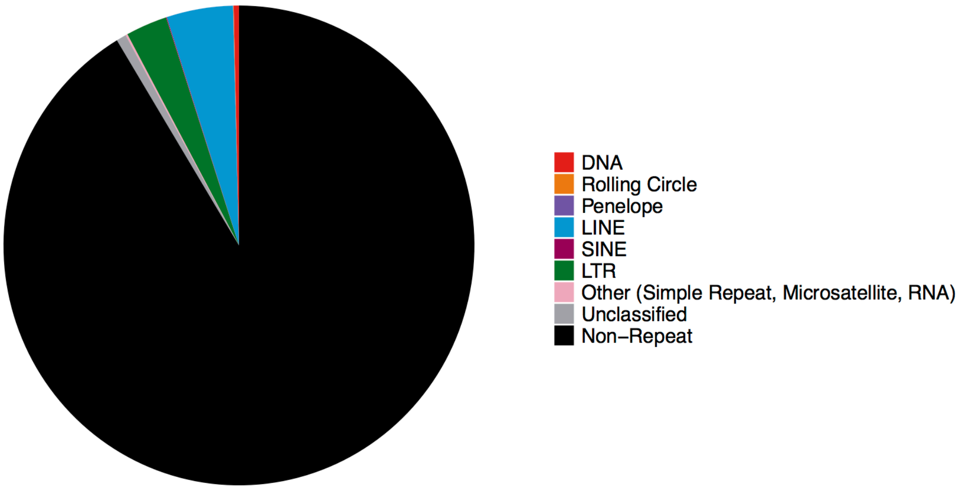

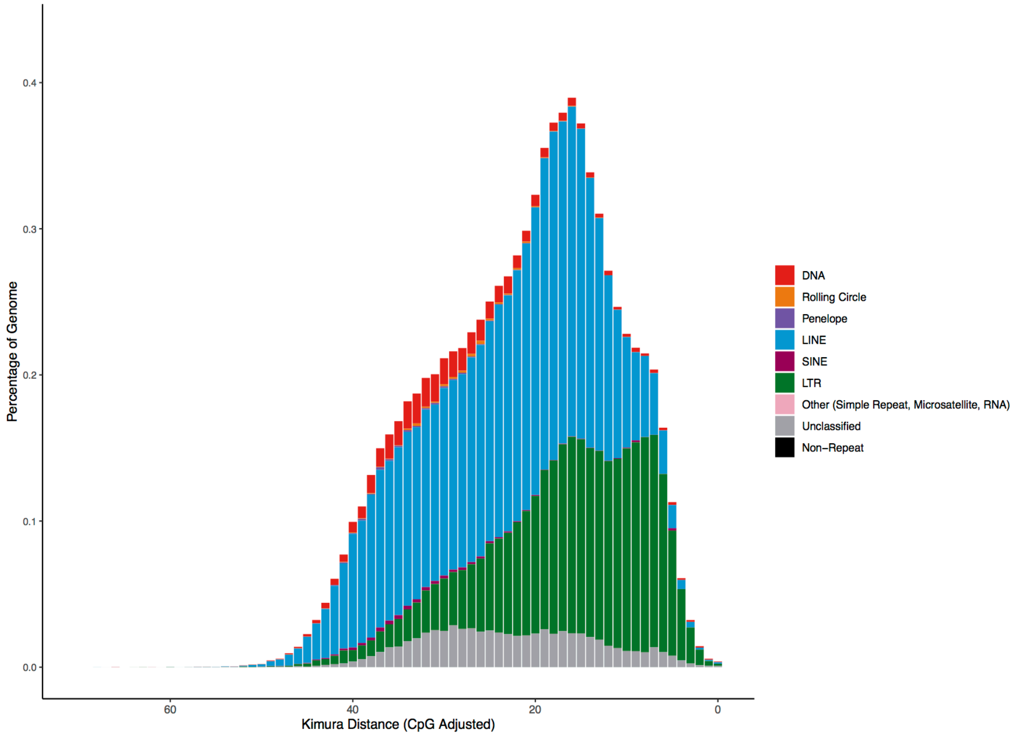

Figure S15. Proportion of repeat elements in *Passer domesticus*, by genome compartment.

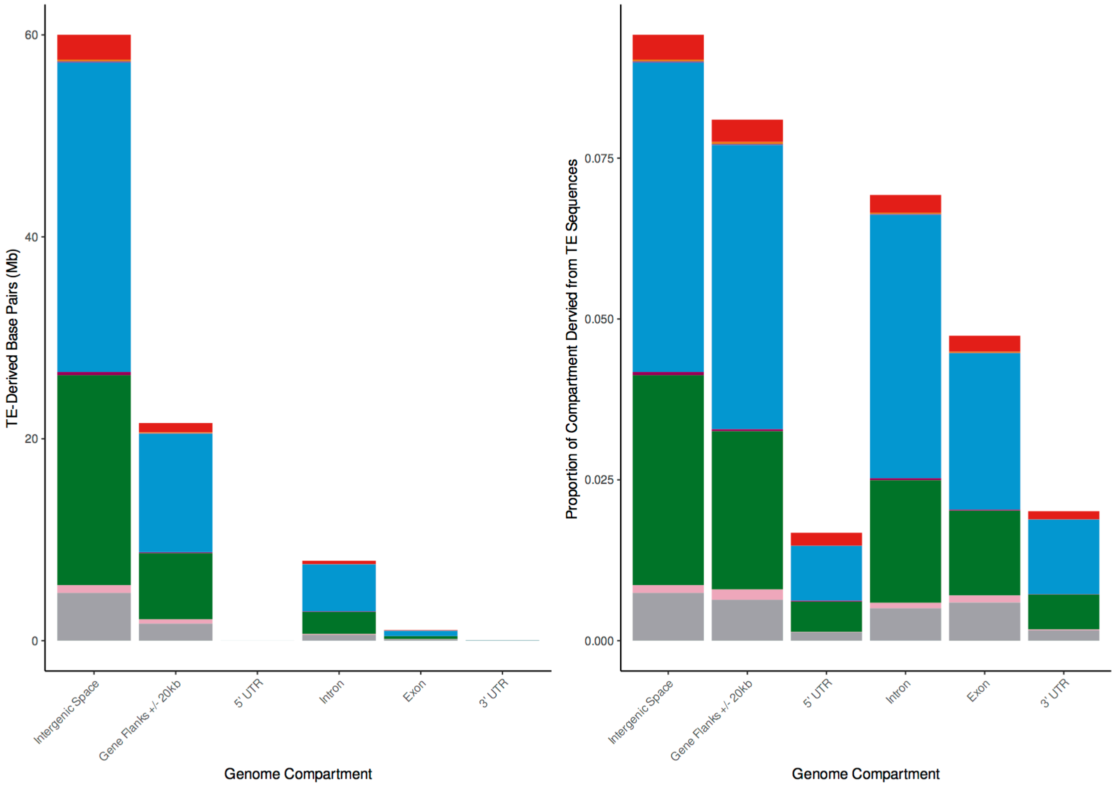

Figure S16. Number of genes at least partially overlapped by 434 curated SVs (retained by the most lenient curator) across the different linkage groups for *Passer domesticus*.

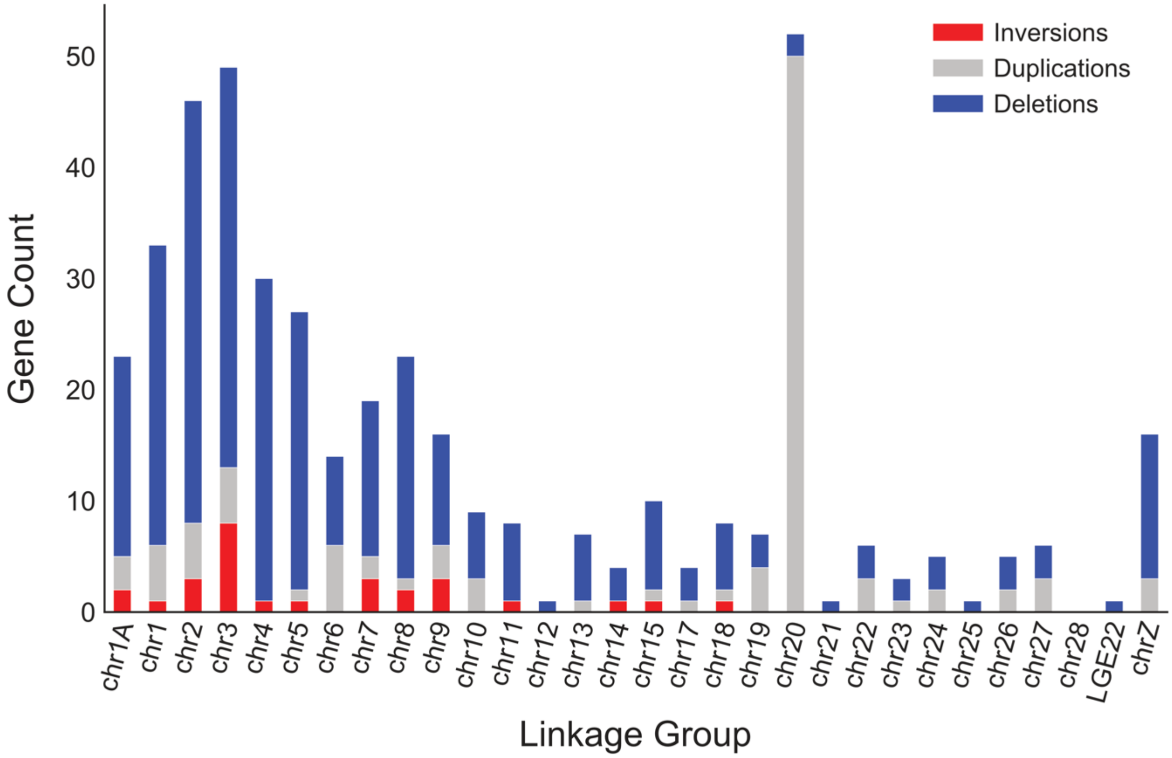

Table S1. % variants removed following filtering by "genotype-frequency", prior to manual curation (SVs selected for curation which are represented by at least three individuals of each genotype class).

Genotype-frequency filtering, uncurated	>20 to 100 bp	>100 to 250 bp	>250 to 500 bp	>500 bp to 1 Kb	>1 to 5 Kb	>5 to 10 Kb	>10 to 500 Kb	>500 Kb	Percent Removed (Total variants removed)
Deletions	**65.3**	**71.2**	**76.5**	**81.4**	**88.5**	**88.8**	**94.4**	**93.9**	**77.0** (11,568)
Duplications	**50.0**	**67.6**	**56.2**	**44.6**	**71.6**	**59.3**	**65.5**	**64.4**	**62.7** (2,149)
Inversions	**66.0**	**85.2**	**81.8**	**88.9**	**97.1**	**91.7**	**97.8**	**94.4**	**78.8** (936)

Table S2. Total SV calls, Putative False Positive Rate and % Agreement between curators. Agreement between Curators = intersect between SVs accepted by strictest curator and other curators.

	DEL	DUP	INV
Single Curator	2457	287	177
% Putative False Positives	29%	78%	30%
2 Curators	1541	45	16
% Putative False Positives	55%	82%	94%
% Agreement btwn Curators	99%	96%	89%
3 Curators	1532	42	15
% Putative False Positives	56%	83%	94%
% Agreement btwn Curators	99%	89%	83%
4 Curators	1243	37	13
% Putative False Positives	64%	97%	95%
% Agreement btwn Curators	80%	79%	72%

Table S3. Mean and Weighted F_{ST} for population structure comparisons in Figure 3. Weighted F_{ST} values are in parentheses.

A. 1,243 DELs curated

	**Trøndelag**	**Finland**	**Pasvik**
**Trøndelag**	-	-	-
**Finland**	0 (0.040)	-	-
**Pasvik**	0.029 (0.116)	0.006 (0.081)	-
**LekaVega**	0.090 (0.178)	0.117 (0.215)	0.159 (0.275)

B. 1,200 random SNPs

	**Trøndelag**	**Finland**	**Pasvik**
**Trøndelag**	-	-	-
**Finland**	0 (0.017)	-	-
**Pasvik**	0.018 (0.073)	0.010 (0.076)	-
**LekaVega**	0.060 (0.123)	0.078 (0.153)	0.126 (0.229)

C. 1,135 DELs rejected by Duphold

	**Trøndelag**	**Finland**	**Pasvik**
**Trøndelag**	-	-	-
**Finland**	0 (0)	-	-
**Pasvik**	0 (0)	0 (0.007)	-
**LekaVega**	0 (0)	0.006 (0.034)	0.008 (0.037)

D. ~30 x 10^6 SNPs

	**Trøndelag**	**Finland**	**Pasvik**
**Trøndelag**	-	-	-
**Finland**	0 (0.028)	-	-
**Pasvik**	0.026 (0.085)	0.018 (0.071)	-
**LekaVega**	0.059 (0.125)	0.075 (0.148)	0.120 (0.208)

E. 1,004 DELs rejected by curator

	Trøndelag	Finland	Pasvik
**Trøndelag**	-	-	-
**Finland**	0 (0)	-	-
**Pasvik**	0 (0)	0 (0.009)	-
**LekaVega**	0 (0.056)	0.012 (0.072)	0.020 (0.092)

Appendix: Identifying Structural Variants with Samplot

Structural Variant: a term that includes large-scale **deletions, duplications,** and **inversions**, usually defined with an arbitrary minimum size greater than 50 base pairs in length (but may be shorter), ranging upwards to hundreds of Megabases (Mb) in length.

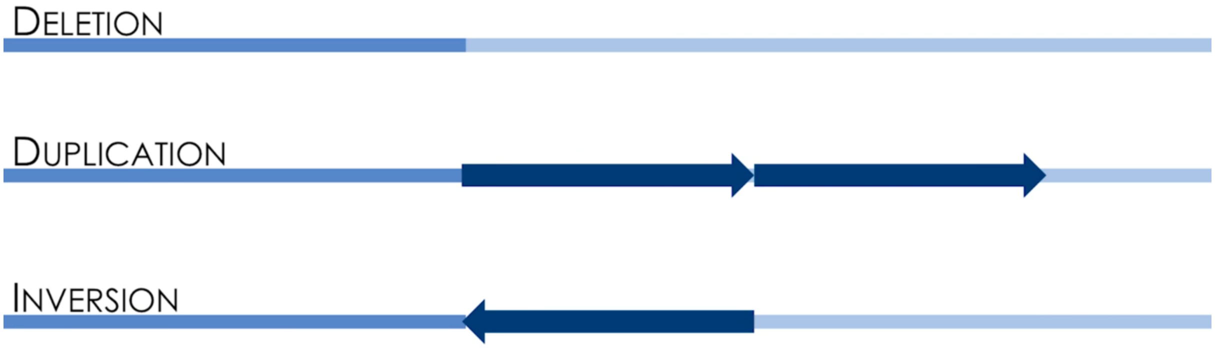

https://github.com/ryanlayer/samplot

Samplot basics
https://www.youtube.com/watch?feature=player_embedded&v=ono8kHMKxDs

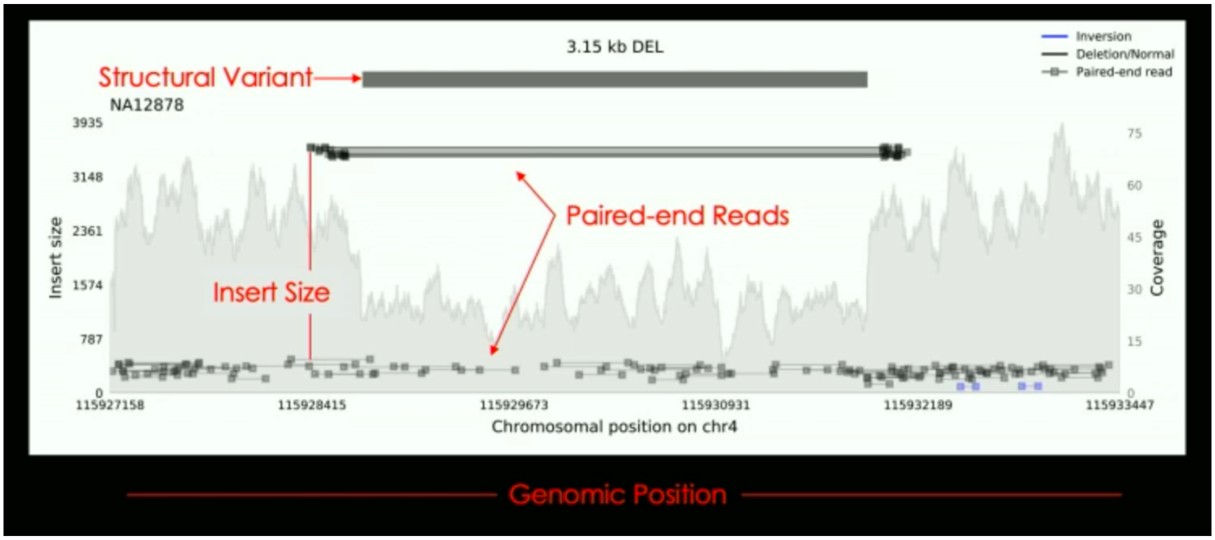

Glossary

DEL: deletion; mutation where a segment of DNA sequence varies in presence/absence relative to the reference genome, can range from a single nucleotide upwards to megabases (Mb) in length.

DUP: duplication; mutation where a segment of DNA sequence is duplicated relative to the reference genome, can range from a single nucleotide upwards to megabases (Mb) in length.

INV: inversion; mutation where a segment DNA sequence is reversed end-to-end (inverted) relative to the reference genome, can range from two nucleotides (2bp) upwards to megabases (Mb) in length.

read-pair: a pair of reads representing a sequenced segment of DNA, with an un-sequenced gap located between the two corresponding pairs, known as the "inner distance" (see Insert size).

Coverage: read depth; **represented as a grey "ridgeline" in Samplot**; number of reads that represent a specific nucleotide or position in the genome.

Insert Size: the total length in nucleotide base pairs, including the two sequenced **read-pairs** (Read 1 and Read 2 below) and the "inner [un-sequenced] distance".

Paired-end Reads (solid lines in Samplot): read pairs where both reads map to the same general location in the genome. For **duplications**, paired-end reads should map in the same location with no consistent overhangs. For **inversions**, paired-end reads should not map in the exact same location, but rather should overlap only in the middle, showing overhangs on both the left and right sides of the overlap. This is indicative of read pairs being reversed or inverted in given genotypes.

Split-reads (dashed lines in Samplot): where one read of a read-pair maps to one location of genome and the other read maps to a different location in a genome; can be one form of supporting evidence for deletions.

Identifying Deletions (DEL), Duplications (DUP) and Inversions (INV)

<u>General steps</u>

Step 1:

First look to see if most **read-pairs**, **Insert size** and change in **Coverage** coincide with the position and length of the Structural Variant, shown at the top of the plot as a thick dark grey bar (**1.39 kb DEL**).

For a putative **“True positive”** homozygote alternate (**1:1**) SV call, the length of **Insert size** should coincide fairly closely with the length SV genotype call specified by the dark grey bar at the top of the plot.

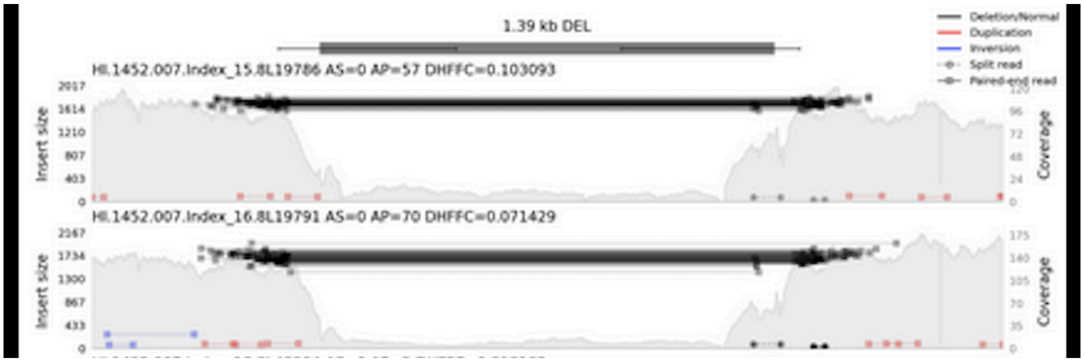

This is a putative “True positive” **deletion**, with read-pairs as black lines squarely overlapping the **1.39 kb DEL** specified as a grey bar at the top of the plot.

Step 2:

Next check to see if the colour of the most common **read-pairs** coincides with the type of SV specified at the top of the plot, according to the legend at the top right:

DELs are black (see example shown above in **Step 1.** for a putative **“True positive” deletion**); **DUPs are red**; and **INVs are blue**.

For example, the following SV calls below are incorrect, showing a **high number of read-pairs** for a type of SV (colour) conflicting with the type of SV call specified by the dark grey bar at the top of the plot:

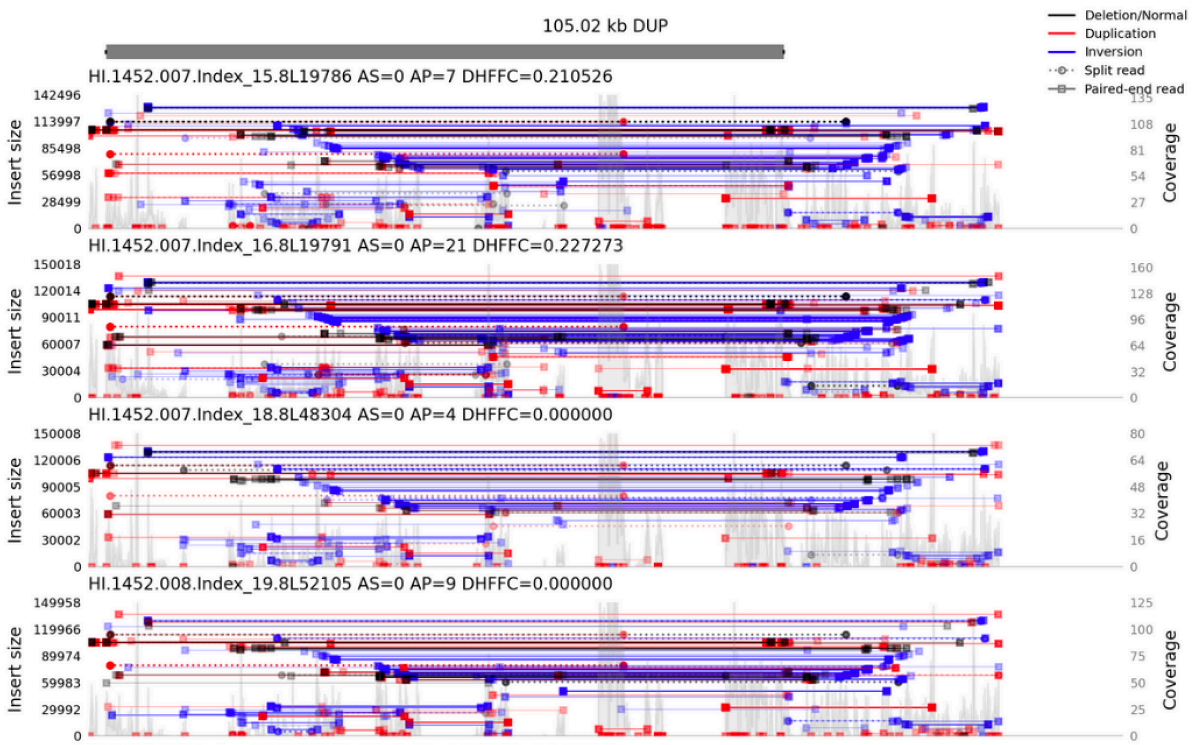

This putative “False positive” **DUP** call shows more read pairs supporting an **INV** in the same region.

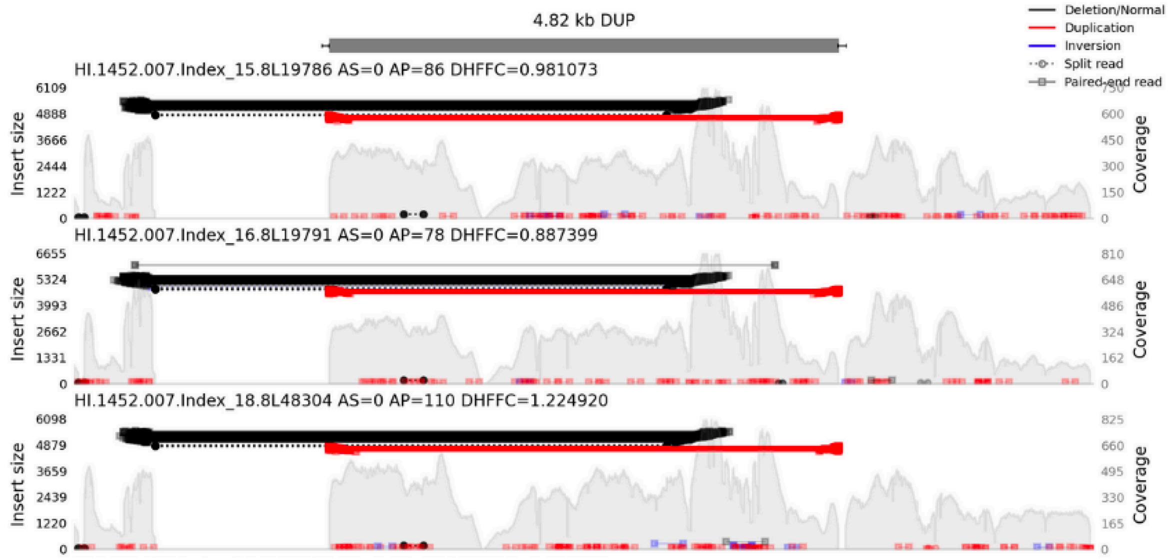

This putative “False positive” **DUP** call shows just as many or more read pairs supporting a **DEL** overlapping the same region.

Step 3:

For **DEL**s and **DUP**s, a major indication of whether the SV call is correct or not comes from the **Coverage** information, represented by the grey ridgeline or mountains fluctuating in the background.

Compare between samples and genotypes. For a real SV call, one would expect variation in the **Coverage** and **read-pair** support between different genotypes: Homozygote for the SV call in question (Homoygote for the alternate allele relative to the reference genome, **1:1**); Heterozygote (**1:0**); Homozygote Wild-Type (**0:0**).

Specific Notes for Identifying Deletions:

-As a first impression, notice that the per-individual genotype calls to left of each plot vary consistently with a striking **Coverage** change between samples.

-Individuals homozygous for **lacking** the **DEL (0:0**; homozygous **wild-type**) show **no change in read depth** within the base pair distance identified by the dark grey bar at the top of the plots.

-The **Insert size** for individuals homozygous alternate (**1:1**) or heterozygous (**1:0**) for the **DEL** should be same size as sequencing insert size, which is the length of the two read pairs plus the "Inner distance": **~150 bp x 2, plus an un-sequenced "Inner distance". See Glossary for definition of terms relating to Insert size.**

- Compare to individuals with other genotypes to note the difference in read-depth (**Coverage**). Individuals with a homozygote alternate genotype (**1:1**) show a clear and sudden drop to zero Coverage in the same distance specified by the dark grey DEL bar at the top of the plot.

-All of the heterozygote genotypes (**0:1**) will show a sudden drop in Coverage, but not completely to zero as in the homozygote alternate.

-Second, both the **0:1** and **1:1** genotypes will show the DEL spanned by a **dashed line**, indicative of a **split read-pair** coinciding with the DEL call. The **wild-type** genotypes (**0:0**) show **no** such **split read-pairs**.

Putative "false positive" Deletion:

-If **all genotypes** show **zero Coverage** within parts of a DEL call, this could just be due to a gap in sequencing for this region of the genome.

-Secondly, there will likely be no split read-pairs straddling the distance indicated by the dark grey DEL bar at the top of the plot.

Specific Notes for Identifying Duplications:

-First notice that individuals that are homozygous wild-type (**0:0**) should show an average **Insert size** for **Paired-end reads** that is the consistent with the normal sequencing Insert size (300 to 400 bp).

-In contrast, homozygous alternate individuals (**1:1**) should show **red bars** indicating **Paired-end reads** of much larger **Insert sizes,** coinciding well with the length **DUP** call shown by the dark grey bar at the top of the plot.

-Next, notice a sudden increase in **Coverage**, creating a noticeably higher "**plateau**" of peaks and a series precisely coinciding with the length of the **DUP** genotype specified by the dark grey bar at the top of the plot.

Putative "False positive" Duplication:

-Firstly, there may be an abrupt break in **Coverage** where the DUP call begins, shown by the dark grey bar at the top of the plot. Regions of zero read-depth or Coverage are cause for suspicion.

-Next, there will likely be mixed signals in terms of the **Insert sizes** of the **Paired-end reads.**

Specific Notes for Identifying Inversions:

High-confidence (putative "True positive") Inversions:

-Unlike Deletions or Duplications, we do not expect a general and obvious change in **Coverage** between individuals with or without a given **Inversion** genotype, because this kind of structural variant should just comprise an end-to-end reversal in orientation.

-However, there is often a slight but noticeably sharp dip in **Coverage** coinciding with the break points on either side of the **INV** genotype call, specified by the length of the dark grey bar at the top of the plot. For example, this sharp **rise and drop in read-depth** can be seen as a narrow peak of Coverage directly next to a narrow "ravine" of Coverage to the left of the INV call length for the homozygote alternate individual (**1:1**), second from the top; to

the right of the INV call length for the heterozygote individual (**0:1**), third from the top; and to the right of the to the right of the INV call length for the homozygote alternate individual (**1:1**), seventh from the top. In contrast, **no such sudden rise and drop in Coverage** can be seen in the **homozygote wild-types (0:0).**

-Next, notice how average **Insert size** hovers around the average sequencing Insert size, as for homozygote wild-type individuals (**0:0**). In contrast, **Insert size** for individuals positive for the INV call show **blue Paired-end reads** and/or **Split-reads** ranging from ~750 bp to ~1100 bp, coinciding fairly well with the length of the INV call specified by the dark grey bar at the top of the plot.

-Next, the **most definitive pattern that a given** INV **call is a putative "true positive"** rather than a "false positive" is the **skewed overlap** of the **read-pairs to the left and right of the break points for the length of the INV call.**

Notice how in homozygote alternate (**1:1**) and heterozygote individuals (**0:1**), all of the **blue Paired-end reads** and/or **Split-reads overlap consistently** within the region of the INV call, specified as a dark grey bar at the top of the plot. However, notice how the reads with an Insert size longer than the INV call extend either to the left or right beyond the break point of the INV call. This alternating **skew in overlap of read-pairs to either side of the INV call** is due to the reversed orientation of reads relative to the homozygous wild-type (0:0) reference individual.

-Finally, homozygous alternate individuals (1:1) generally show a denser accumulation of **blue Paired-end reads** and/or **Split-reads in skewed overlap** than heterozygous individuals (0:1)—but this may not be a definitive difference.

Putative "false positive" Inversion:

-As with the putative false positive Duplication, notice the **complete absence of Coverage** in some parts of the INV call, a cause for suspicion. In addition, there is often a **wild fluctuation in Coverage** along the length of the INV call.

-Notice also how there is little variation in the density of **blue Paired-end reads** between homozygote wild-types (**0:0**), heterozygotes (**0:1**) and homozygote alternates (**1:1**).

Example of high-confidence putative True and False positive DELs, DUPs and INVs from this study and of:

Bertolotti, Alicia C., Ryan M. Layer, Manu Kumar Gundappa, Michael D. Gallagher, Ege Pehlivanoglu, Torfinn Nome, Diego Robledo et al. **The structural variation landscape in 492 Atlantic salmon genomes**. *Nature Communications* 11, no. 1 (2020): 5176. (supplementary figures reproduced with permission from the authors, under the Creative Commons license: http://creativecommons.org/licenses/by/4.0/)

Example 1:

Example putative "True positive" **DEL** from ***Passer domesticus*** SV calls:

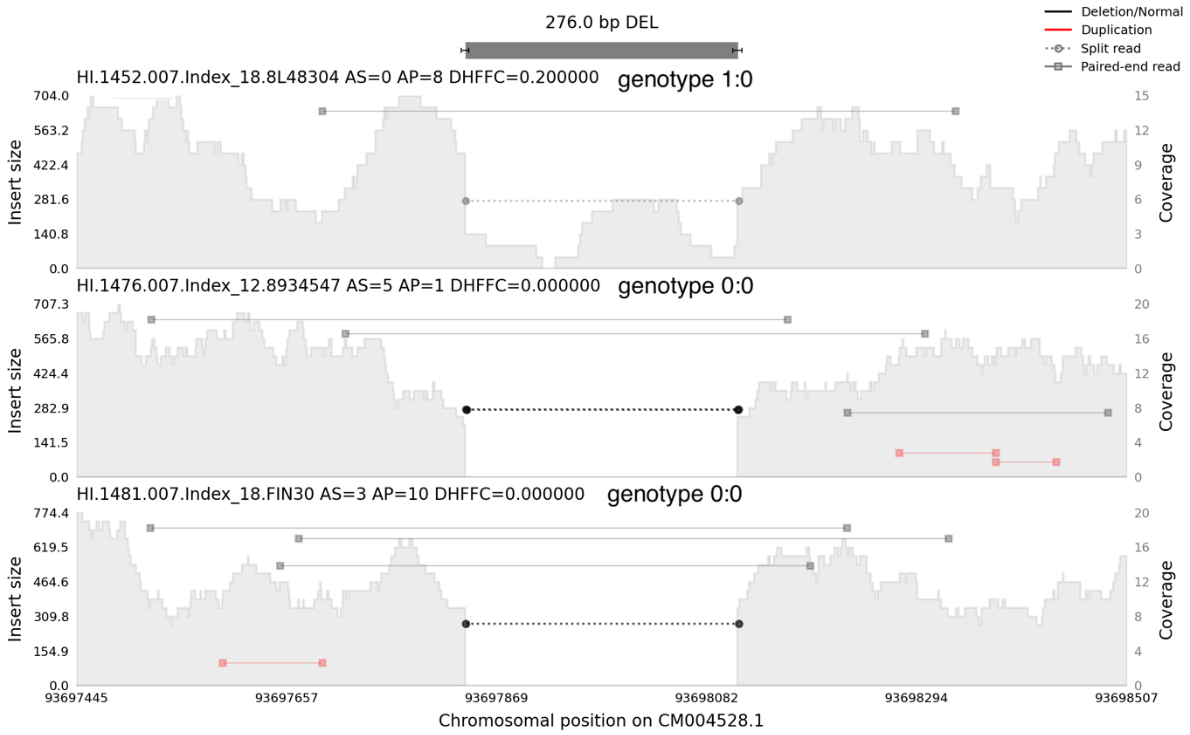

Notice the intermediate coverage in the heterozygote (**1:0**) and a gap straddled by a split-read signal in the homozygotes (**1:1**) which overlap the length of the SV specified by the grey bar at the top of the plot (**276.0 bp DEL**).

High-confidence (putative "True positive") Deletion:

-As a first impression, notice that the per-individual genotype calls to left of each plot vary consistently with a striking **Coverage** change between samples.

-Individuals homozygous for **lacking** the **DEL (0:0**; homozygous **wild-type**) show no change in read depth within the base pair distance identified by the dark grey bar at the top of the plots (**360.0 bp <DEL>**).

- Compare to individuals with other genotypes to note the difference in read-depth (**Coverage**). Individuals with a homozygote alternate genotype (**1:1**) show a clear and sudden drop to zero Coverage in the same distance specified by the dark grey DEL bar at the top of the plot.

-All of the heterozygote genotypes (**0:1**) show a sudden drop in Coverage, but not completely to zero as in the homozygote alternate.

-Second, notice that both the **0:1** and **1:1** genotypes show the DEL spanned by a **dashed line**, indicative of a **split read-pair** coinciding with the DEL call. The **wild-type** genotypes (**0:0**) show **no** such **split read-pairs**.

-In this case, the **red colour** coinciding to a DUP is **less important** and can be disregarded in comparison to the other strong evidence for the DEL, which varies consistently between different genotype calls.

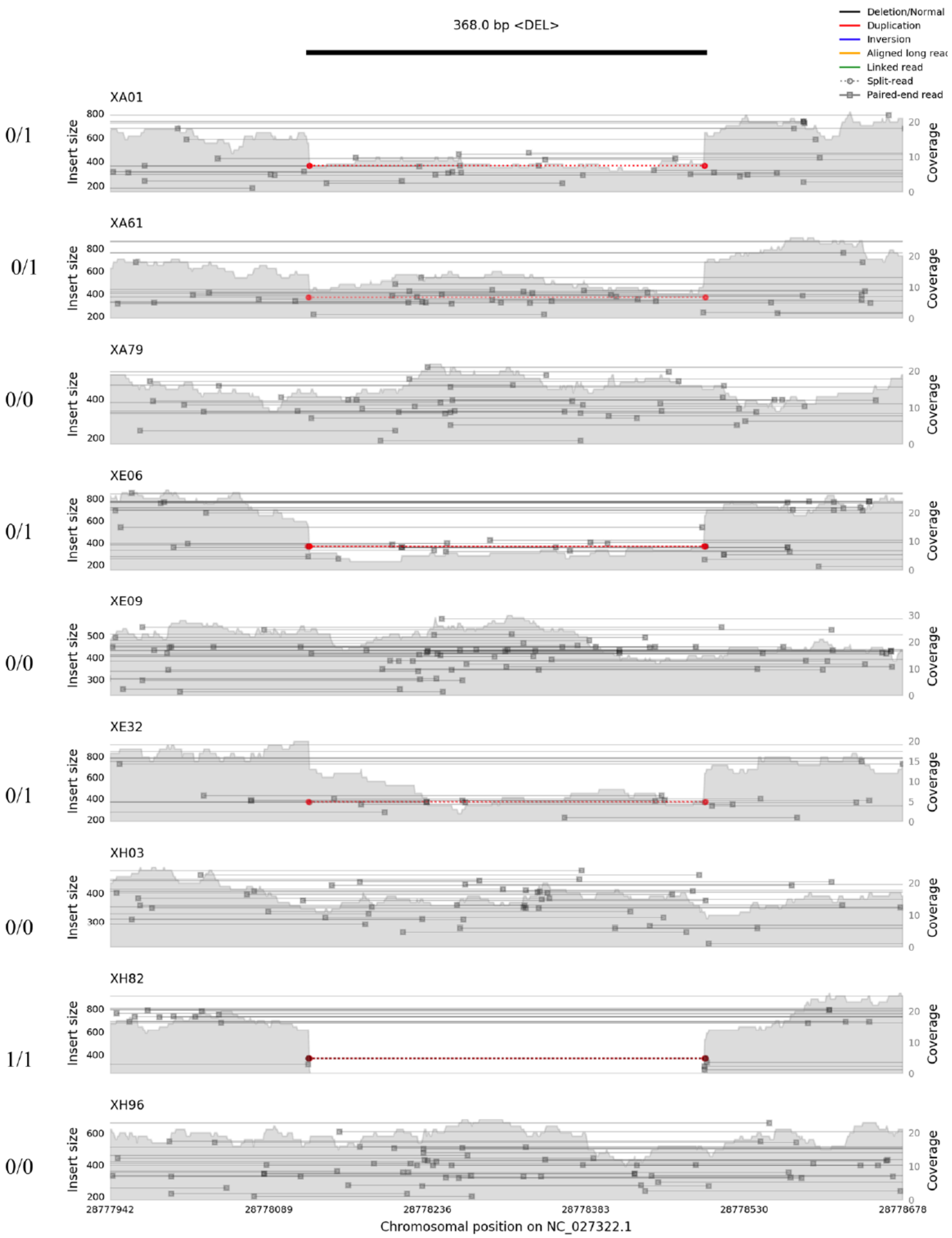
368.0 bp <DEL>
Deletion/Normal
Duplication
Inversion
Aligned long read
Linked read
Split-read
Paired-end read
XA01
0/1
XA61
0/1
XA79
0/0
XE06
0/1
XE09
0/0
XE32
0/1
XH03
0/0
XH82
1/1
XH96
0/0
Insert size
Coverage
28777942
28778089
28778236
28778383
28778530
28778678
Chromosomal position on NC_027322.1

Putative "false positive" Deletion:

-As a first impression, **all genotypes** show **zero Coverage** within parts of the DEL call. This could just be due to a gap in sequencing for this region of the genome.

-Secondly, there are no split read-pairs straddling the distance indicated by the dark grey DEL bar at the top of the plot (819.0 bp <DEL>).

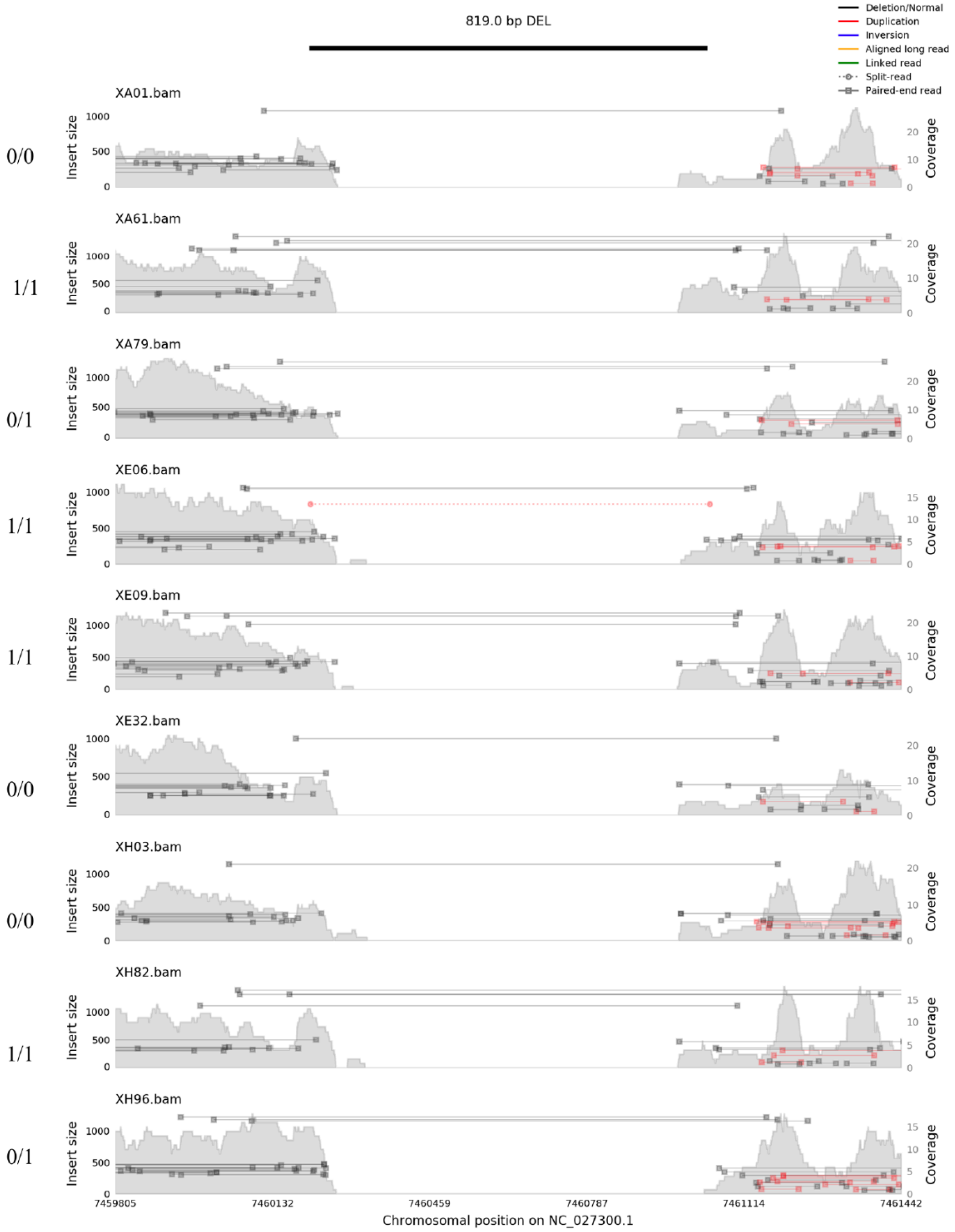
819.0 bp DEL
Deletion/Normal
Duplication
Inversion
Aligned long read
Linked read
Split-read
Paired-end read
XA01.bam
0/0
XA61.bam
1/1
XA79.bam
0/1
XE06.bam
1/1
XE09.bam
1/1
XE32.bam
0/0
XH03.bam
0/0
XH82.bam
1/1
XH96.bam
0/1
Insert size
Coverage
7459805
7460132
7460459
7460787
7461114
7461442
Chromosomal position on NC_027300.1

High-confidence (putative "True-positive") Duplication:

-Notice that individuals that are homozygous wild-type (**0:0**) show an average **Insert size** for **Paired-end reads** around ~**400 bp**.

-In contrast, homozygous alternate individuals (**1:1**) show **red bars** indicating **Paired-end reads** of much larger **Insert size** around ~**20,000 bp,** which coincides well with the length **DUP** call shown by the dark grey bar at the top of the plot (**21.52 kb <DUP>)**.

-Next, notice a sudden increase in **Coverage,** in this case almost double from a read-depth of 20 up to 40, creating a noticeably higher "**plateau**" of peaks and a series precisely coinciding with the length of the **DUP** genotype specified by the dark grey bar at the top of the plot (**21.52 kb <DUP>)**. The bottommost individual genotyped as homozygote alternate (**1:1**) is less clear than the other two **1:1** genotypes above, but nonetheless shows some strikingly higher peaks within the length specified by the DUP call.

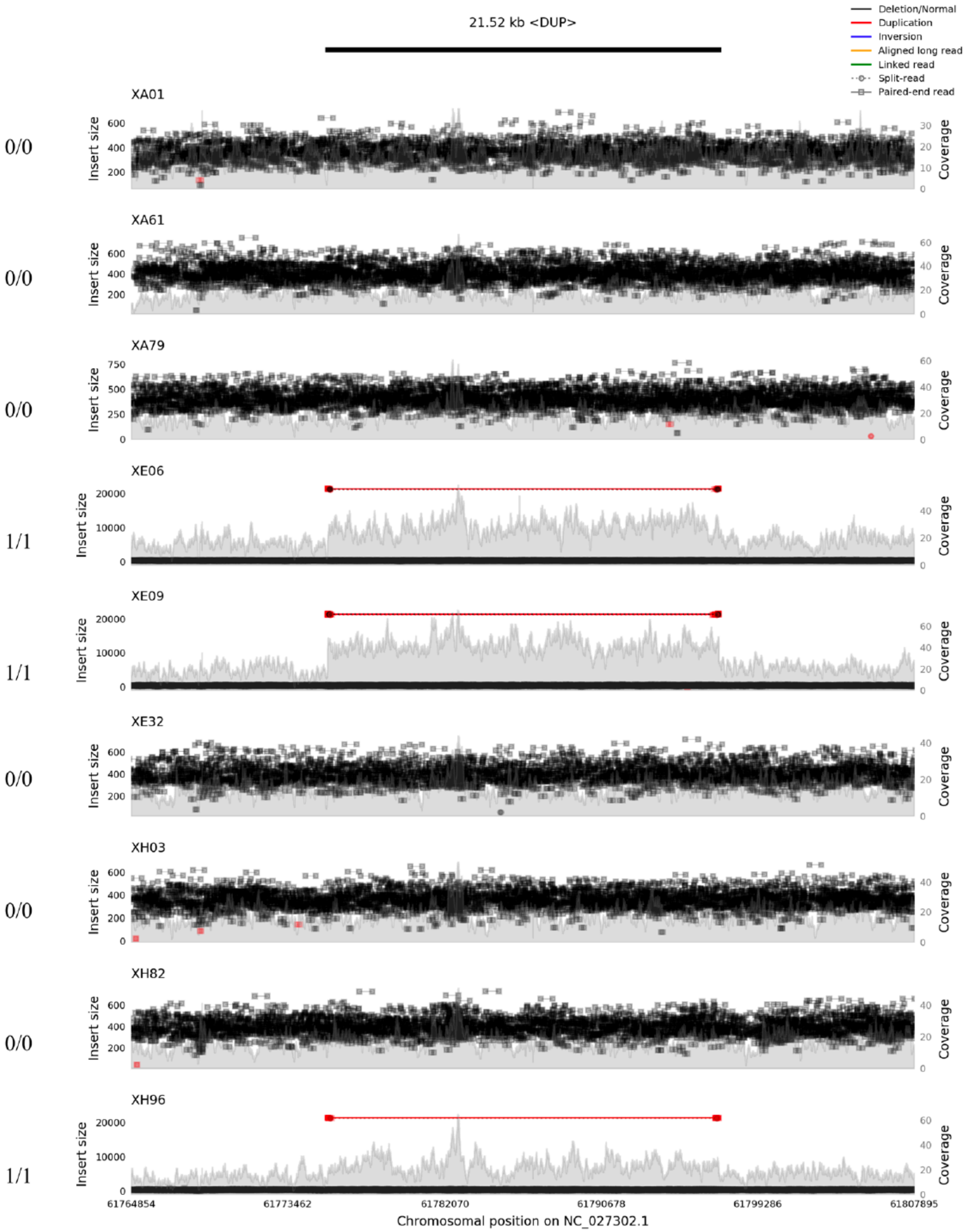
21.52 kb <DUP>
Deletion/Normal
Duplication
Inversion
Aligned long read
Linked read
Split-read
Paired-end read
XA01
0/0
XA61
0/0
XA79
0/0
XE06
1/1
XE09
1/1
XE32
0/0
XH03
0/0
XH82
0/0
XH96
1/1
Insert size
Coverage
61764854
61773462
61782070
61790678
61799286
61807895
Chromosomal position on NC_027302.1

Putative "false positive" Duplication:

-Firstly, notice the abrupt break in **Coverage** where the DUP call begins, shown by the dark grey bar at the top of the plot. Regions of zero read-depth or Coverage are cause for suspicion.

-Next, notice that there are mixed signals in terms of the **Insert sizes** of the **Paired-end reads.** Even though some **Paired-ends reads** do increase significantly to ~3000 bp in **Insert size** within the length of the DUP call (**2.76 Kb <DUP>),** these are contradicted by much smaller, average **Insert sizes** of ~400 bp **within the same individual**.

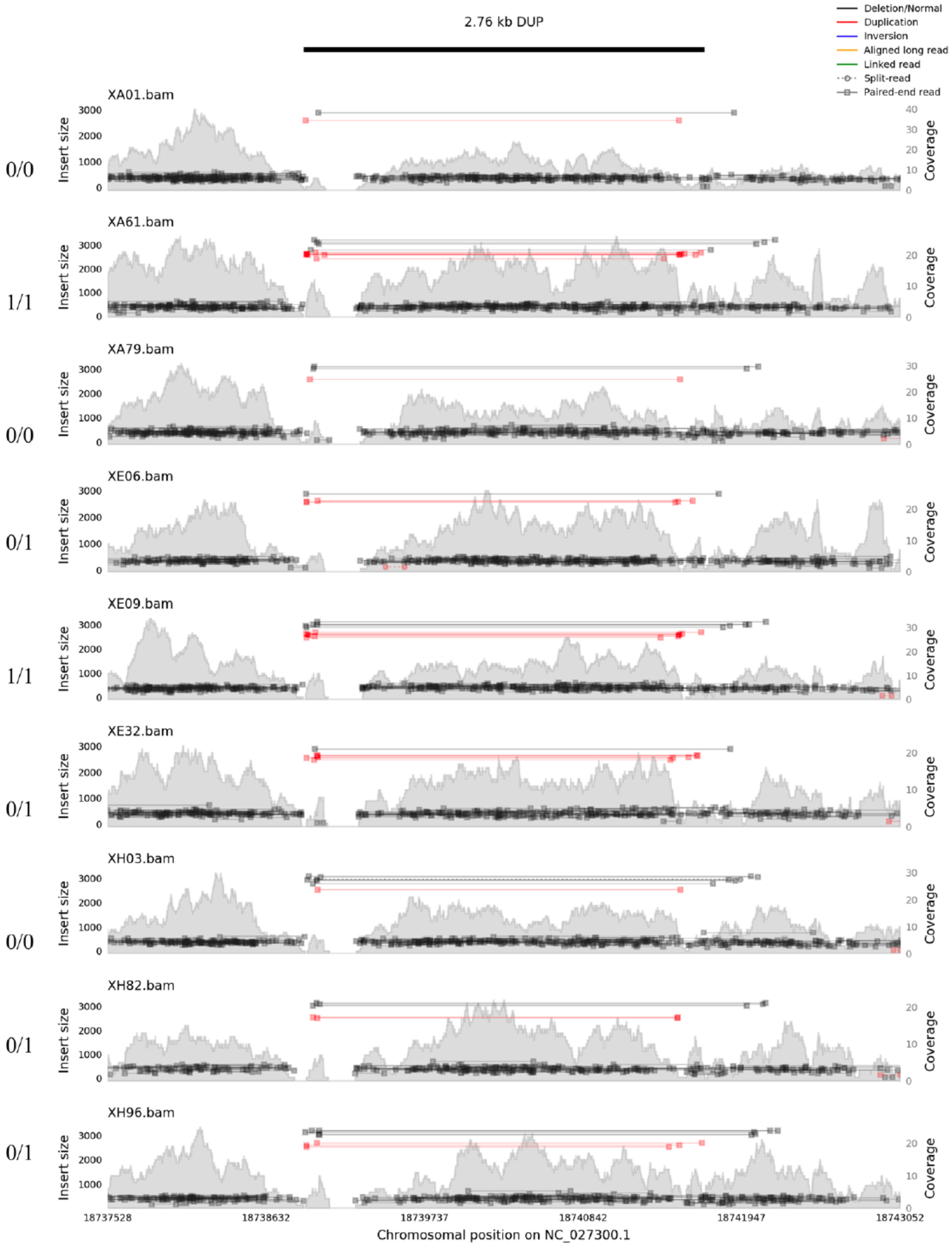
2.76 kb DUP
Deletion/Normal
Duplication
Inversion
Aligned long read
Linked read
Split-read
Paired-end read
XA01.bam
0/0
XA61.bam
1/1
XA79.bam
0/0
XE06.bam
0/1
XE09.bam
1/1
XE32.bam
0/1
XH03.bam
0/0
XH82.bam
0/1
XH96.bam
0/1
Insert size
Coverage
18737528
18738632
18739737
18740842
18741947
18743052
Chromosomal position on NC_027300.1

Specific Notes for Identifying Inversions:

High-confidence (putative "True positive") Inversions:

-Unlike Deletions or Duplications, we do not expect a general and obvious change in **Coverage** between individuals with or without a given **Inversion** genotype, because this kind of structural variant should just comprise an end-to-end reversal in orientation.

-However, there is often a slight but noticeably sharp dip in **Coverage** coinciding with the break points on either side of the **INV** genotype call, specified by the length of the dark grey bar at the top of the plot. For example, this sharp **rise and drop in read-depth** can be seen as a narrow peak of Coverage directly next to a narrow "ravine" of Coverage to the left of the INV call length for the homozygote alternate individual (**1:1**), second from the top; to the right of the INV call length for the heterozygote individual (**0:1**), third from the top; and to the right of the to the right of the INV call length for the homozygote alternate individual (**1:1**), seventh from the top. In contrast, **no such sudden rise and drop in Coverage** can be seen in the **homozygote wild-types (0:0).**

-Next, notice how average **Insert size** hovers around an average **~400 bp** for homozygote wild-type individuals (**0:0**). In contrast, **Insert size** for individuals positive for the INV call show **blue Paired-end reads** and/or **Split-reads** ranging from ~750 bp to ~1100 bp, coinciding fairly well with the length of the INV call specified by the dark grey bar at the top of the plot **(903.0 bp <INV>)**.

-Next, the **most definitive pattern that a given INV call is a putative "true positive"** rather than a putative "false positive" is the **skewed overlap** of the **read-pairs to the left and right of the break points for the length of the INV call:**

Notice how in homozygote alternate (**1:1**) and heterozygote individuals (**0:1**), all of the **blue Paired-end reads** and/or **Split-reads overlap consistently** within the region of the INV call, specified as a dark grey bar at the top of the plot **(903.0 bp <INV>)**. However, notice how the reads with an Insert size longer than the INV call extend either to the left or right beyond the break point of the INV call. This alternating **skew in overlap of read-pairs to either side of the INV call** is due to the reversed orientation of reads relative to the homozygous wild-type (0:0) reference individual.

-Finally, homozygous alternate individuals (1:1) generally show a denser accumulation of **blue Paired-end reads** and/or **Split-reads in skewed overlap** than heterozygous individuals (0:1)—but this may not be a definitive difference.

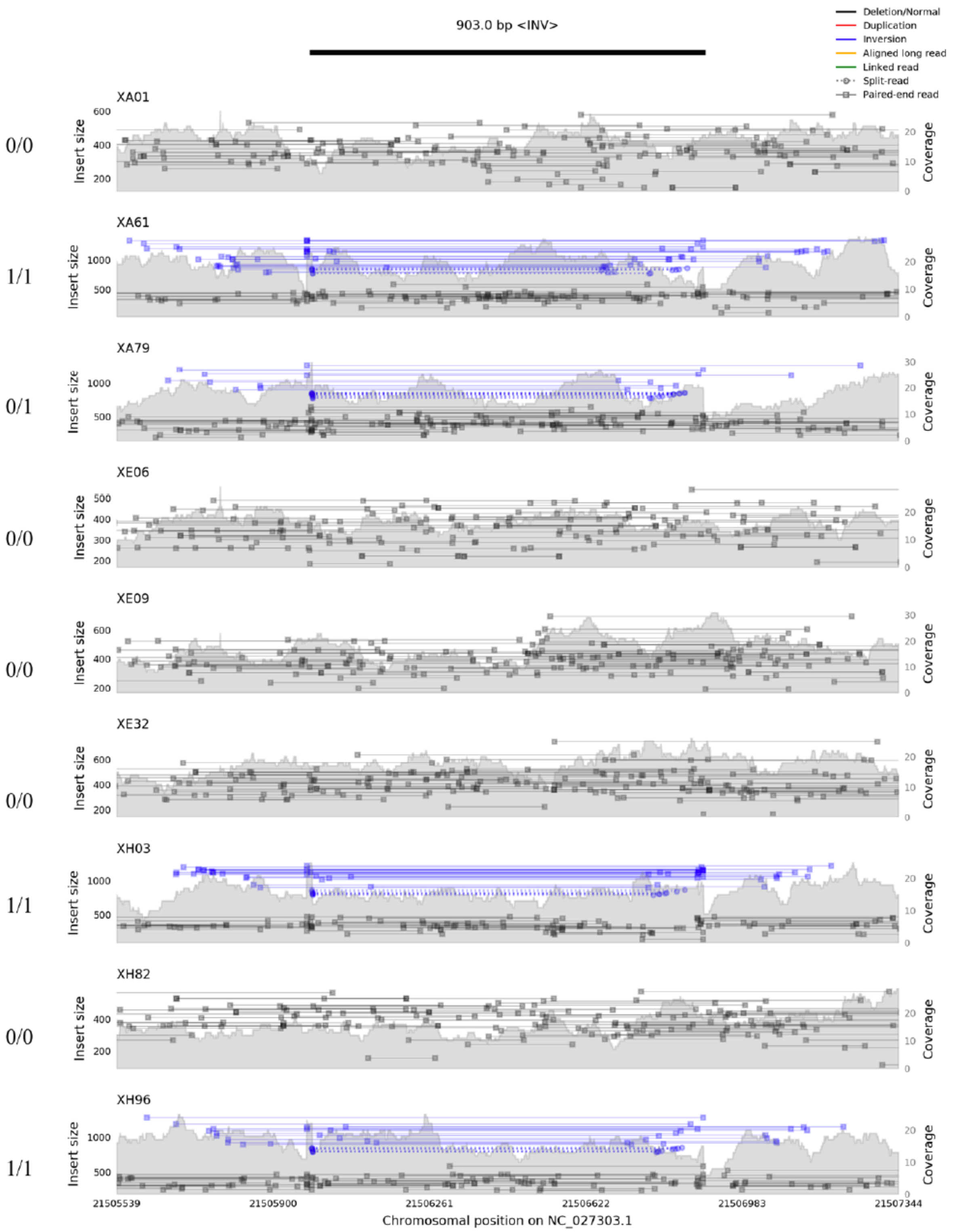

903.0 bp <INV>
Deletion/Normal
Duplication
Inversion
Aligned long read
Linked read
Split-read
Paired-end read
XA01
0/0
XA61
1/1
XA79
0/1
XE06
0/0
XE09
0/0
XE32
0/0
XH03
1/1
XH82
0/0
XH96
1/1
Insert size
Coverage
21505539
21505900
21506261
21506622
21506983
21507344
Chromosomal position on NC_027303.1

Example 2:

Example putative **“True positive”** **INV** from ***Passer domesticus*** SV calls:

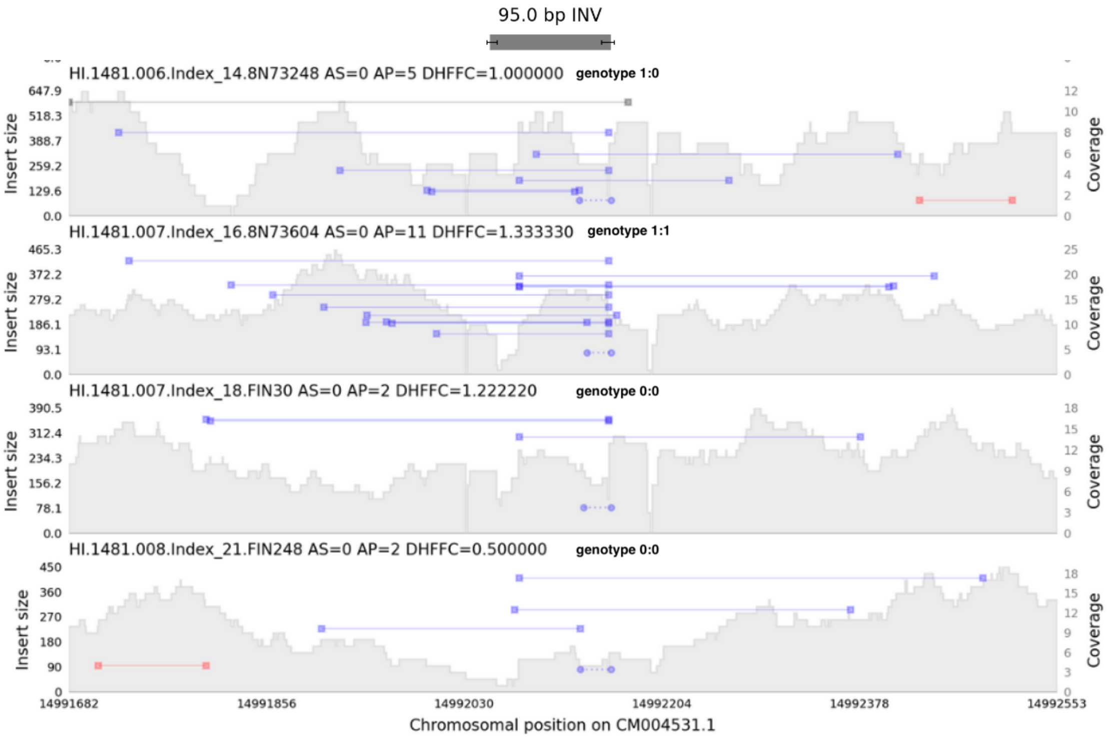

Notice the overhangs particularly pronounced in the homozygote alternate and the marked dip in coverage on either side of the inversion, suggesting the inversion breakpoints.

Putative “false positive” Inversion:

-As with the putative “false positive” Duplication, notice the **complete absence of Coverage** in some parts of the INV call, a cause for suspicion. In addition, there is a **wild fluctuation in Coverage** from 1000 bp to 0 bp along the length of the INV call.

-Notice also how there is little variation in the density of **blue Paired-end reads** between homozygote wild-types (**0:0**), heterozygotes (**0:1**) and homozygote alternates (**1:1**).

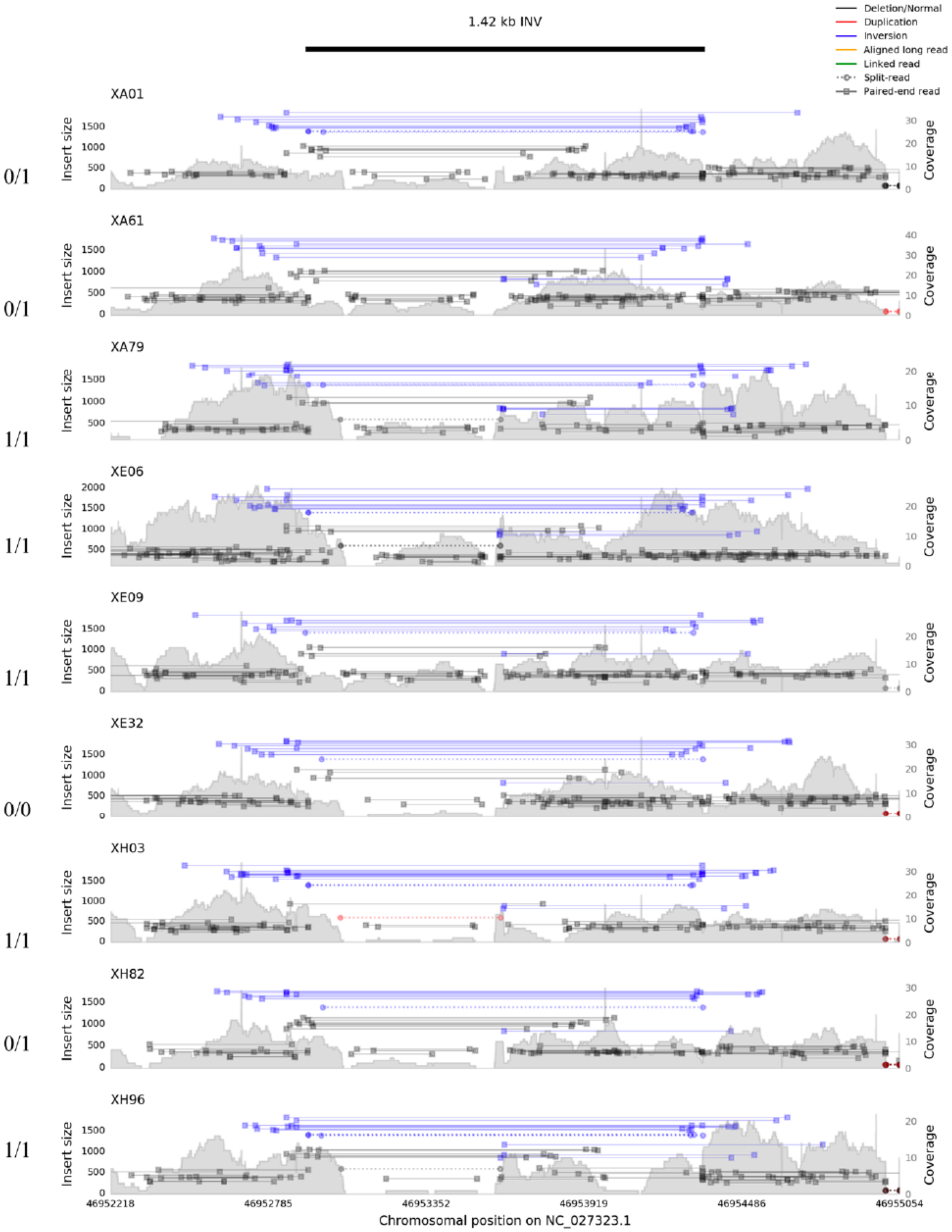
1.42 kb INV
Deletion/Normal
Duplication
Inversion
Aligned long read
Linked read
Split-read
Paired-end read
XA01
0/1
XA61
0/1
XA79
1/1
XE06
1/1
XE09
1/1
XE32
0/0
XH03
1/1
XH82
0/1
XH96
1/1
Insert size
Coverage
46952218
46952785
46953352
46953919
46954486
46955054
Chromosomal position on NC_027323.1
